# Refining and illuminating acetogenic *Eubacterium* strains for reclassification and metabolic engineering

**DOI:** 10.1186/s12934-024-02301-8

**Published:** 2024-01-17

**Authors:** Maximilian Flaiz, Anja Poehlein, Wiebke Wilhelm, Alexander Mook, Rolf Daniel, Peter Dürre, Frank R. Bengelsdorf

**Affiliations:** 1 Laboratory of Microbiology, Wageningen University and Research, Wageningen, The Netherlands; 2 Institute of Molecular Biology and Biotechnology of Prokaryotes, University of Ulm, Ulm, Germany; 3 Genomic and Applied Microbiology & Göttingen Genomics Laboratory, Georg-August University, Göttingen, Germany; 4 Institute of Microbiology and Biotechnology, University of Ulm, Ulm, Germany

**Keywords:** Acetogens, Anaerobes, callanderi, *Eubacterium*, FAST, Fluorescence, Hexanoate, limosum, maltosivorans, Methanol

## Abstract

**Background:**

The genus *Eubacterium* is quite diverse and includes several acetogenic strains capable of fermenting C1-substrates into valuable products. Especially, *Eubacterium limosum* and closely related strains attract attention not only for their capability to ferment C1 gases and liquids, but also due to their ability to produce butyrate. Apart from its well-elucidated metabolism, *E. limosum* is also genetically accessible, which makes it an interesting candidate to be an industrial biocatalyst.

**Results:**

In this study, we examined genomic, phylogenetic, and physiologic features of *E. limosum* and the closest related species *E. callanderi* as well as *E. maltosivorans*. We sequenced the genomes of the six *Eubacterium* strains ‘FD’ (DSM 3662^T^), ‘Marburg’ (DSM 3468), ‘2A’ (DSM 2593), ‘11A’ (DSM 2594), ‘G14’ (DSM 107592), and ‘32’ (DSM 20517) and subsequently compared these with previously available genomes of the *E. limosum* type strain (DSM 20543^T^) as well as the strains ‘B2’, ‘KIST612’, ‘YI’ (DSM 105863^T^), and ‘SA11’. This comparison revealed a close relationship between all eleven *Eubacterium* strains, forming three distinct clades: *E. limosum*, *E. callanderi*, and *E. maltosivorans*. Moreover, we identified the gene clusters responsible for methanol utilization as well as genes mediating chain elongation in all analyzed strains. Subsequent growth experiments revealed that strains of all three clades can convert methanol and produce acetate, butyrate, and hexanoate via reverse β-oxidation. Additionally, we used a harmonized electroporation protocol and successfully transformed eight of these *Eubacterium* strains to enable recombinant plasmid-based expression of the gene encoding the fluorescence-activating and absorption shifting tag (FAST). Engineered *Eubacterium* strains were verified regarding their FAST-mediated fluorescence at a single-cell level using a flow cytometry approach. Eventually, strains ‘FD’ (DSM 3662^T^), ‘2A’ (DSM 2593), ‘11A’ (DSM 2594), and ‘32’ (DSM 20517) were genetically engineered for the first time.

**Conclusion:**

Strains of *E. limosum*, *E. callanderi*, and *E. maltosivorans* are outstanding candidates as biocatalysts for anaerobic C1-substrate conversion into valuable biocommodities. A large variety of strains is genetically accessible using a harmonized electroporation protocol, and FAST can serve as a reliable fluorescent reporter protein to characterize genetically engineered cells. In total eleven strains have been assigned to distinct clades, providing a clear and updated classification. Thus, the description of respective *Eubacterium* species has been emended, improved, aligned, and is requested to be implemented in respective databases.

**Supplementary Information:**

The online version contains supplementary material available at 10.1186/s12934-024-02301-8.

## Background

The conversion of greenhouse gases into valuable products using acetogenic bacteria is an auspicious attempt to combat the ongoing climate crisis. Acetogens convert various C1-carbon sources such as CO, CO_2_, methanol, or formate via the Wood-Ljungdahl pathway (WLP), which is known to be the energetically most efficient C1 fixation pathway [1]. These anaerobic bacteria form a diverse group of bacteria including species of the genera *Acetobacterium*, *Clostridium*, *Eubacterium*, *Morella*, *Sporomusa*, or *Thermoanaerobacter* just to mention a few [2]. While most acetogens produce acetate as the main metabolic product, some of them, i.e. *Eubacterium limosum*, are capable of producing higher, more-valuable compounds such as butyrate or hexanoate [3].

The genus *Eubacterium* was proposed in 1938 by Prévot and the description covers Gram-positive, obligate anaerobic, non-spore-forming rod-shaped bacteria [4]. This uncommitted definition of the genus also applies to a whole range of other bacteria, not belonging to *Eubacterium* sensu stricto [5]. The type species of this genus is *E. limosum* (DSM 20543^T^ = ATCC 8486^T^ = NCIB 9763^T^) and was isolated in 1935 as ‘*Bacteroides limosus*’ from human feces and validly described in 1938 [6]. Recently, *E. limosum* attracted attention as potential biocatalyst, due to its ability to grow chemolithotrophically utilizing gaseous and liquid C1 carbon sources, while producing acetate, butyrate, hexanoate, and low amounts of butanol natively [7]. Moreover, the type strain of *E. limosum* (DSM 20543^T^) is genetically accessible with some available molecular tools [8–11]. The respective genome was sequenced and is publicly available [12]. Over the last decades, several strains related to *E. limosum* were isolated and often treated synonymously. These entail the strains ‘32’ (DSM 20517), ‘RF’, ‘B2’, and ‘KIST612’ [13–16]. However, these strains differ from each other when comparing their nowadays available genomic, morphologic, physiologic, and phylogenetic features. Strain ‘32’ (DSM 20517) was initially described as ‘*Butyribacterium rettgeri*’ and proposed as the first type species of the genus *Butyribacterium* [13]. However, strains ‘32’ (DSM 20517) and the type strain of *E. limosum* (DSM 20543^T^) were shown to be equal regarding cell morphology and physiological properties [17]. Even more persuasive seemed the previous finding, that the comparative cataloging of 16S rRNA oligonucleotides was identical for both strains [18]. Although strain ‘32’ (DSM 20517) is still publicly available, it has never been confirmed to be identical to the type strain of *E. limosum* (DSM 20543^T^) based on genomic data. Strains ‘RF’ and ‘B2’ were isolated in the 1980s and their ability to utilize CO and methanol was meticulously examined [15, 16]. Just recently, the genome of ‘B2’ (in our previous study referred to as ‘NG-6894’) was published and we showed that the strain is genetically accessible [19, 20]. Likewise, the strain ‘KIST612’ was investigated extensively. Studies included analysis regarding the C1 metabolism, genome sequencing, and the establishment of various genetic tools [21–23]. Although ‘KIST612’ was initially described as *E. limosum*, the strain was recently considered to be an *E. callanderi* strain due to the high sequence similarity when compared to the respective type strain [24]. The type strain of *E. callanderi* (strain ‘FD’, DSM 3662^T^) is physiologically highly similar when compared to *E. limosum*. However, Mountfort and co-authors claimed that strain ‘FD’ (DSM 3662^T^) cannot utilize C1 carbon sources [25, 26]. Next, the strain ‘Marburg’ (DSM 3468) originally termed ‘*Butyribacterium methylotrophicum*’ was considered to be reclassified as *Eubacterium*, due to its high genome similarity with *E. limosum* [27, 28]. Its ability to form atypical spores caused the classification of the isolated strain ‘Marburg’ (DSM 3468) as a new genus, which was never officially published according to the rules of the ‘International Code of Nomenclature of Bacteria’ (Bacteriological Code) of the ‘International Committee on Systematics of Prokaryotes’. Strain ‘Marburg’ (DSM 3468) gained interest due to its ability to produce butanol naturally when adapted to CO [29]. Moreover, it was shown that this strain is also a potent methanol utilizer, it is genetically accessible, and first genetic tools were established [30, 31]. Further closely related *Eubacterium* strains were deposited in strain collections, their genome sequenced or just described in the literature. However, a clear allocation of these *Eubacterium* strains is missing.

In this work, the phylogenetic and physiologic characteristics of the following eleven *Eubacterium* strains were compared: DSM 20543^T^, ‘B2’, ‘FD’ (DSM 3662^T^), ‘Marburg’ (DSM3468), ‘KIST612’, ‘2A’ (DSM 2593), ‘11A’ (DSM 2594), ‘G14’ (DSM 107592), ‘YI’ (DSM 105863^T^), ‘32’ (DSM 20517), and ‘SA11’. Therefore, the genomes of six strains (‘FD’ (DSM 3662^T^), ‘Marburg’ (DSM3468), ‘2A’ (DSM 2593), ‘11A’ (DSM 2594), ‘G14’ (DSM 107592), and ‘32’ (DSM 20517)) were sequenced. Analysis of the sequenced genomes revealed that all strains belong to the genus *Eubacterium* and can be categorized into three distinct clades, which are assigned to the type strains of *E. limosum* (DSM 20543^T^), *E. callanderi* (DSM 3662^T^), and *E. maltosivorans* (DSM 105863^T^). Thus, some of these strains had to be reclassified. Representatives of all three clades were analyzed focusing on methanol utilization and their capability of producing hexanoate via chain elongation. Moreover, we emphasized on the establishment of a harmonized electroporation protocol for eight of these strains ((DSM 20543^T^), ‘B2’, ‘FD’ (DSM 3662^T^), ‘Marburg’ (DSM3468), ‘KIST612’, ‘2A’ (DSM 2593), ‘11A’ (DSM 2594), and ‘32’ (DSM 20517)) and used the fluorescence-activating and absorption shifting tag (FAST) as reporter protein to prove successful strain engineering. The presented experiments represent the basis towards the sustainable natural or recombinant production of biocommodities with methanol-utilizing *Eubacterium* species.

## Materials and methods

### Strains, medium, and cultivation

*Eubacterium* strains used in this study and respective strain collection numbers are listed in Table 1. Strains DSM20543^T^, DSM 3662^T^, DSM 3468, DSM 2593, DSM 2594, DSM 107592, and DSM 20517 were purchased from DSMZ (Leibniz-Institut DSMZ-Deutsche Sammlung von Mikroorganismen und Zellkulturen GmbH, Brunswick, Germany). Strains ‘B2’ and ‘KIST612’ were kindly provided by Phillipe Soucaille (INSA, UPS, INP, Toulouse Biotechnology Institute, Université de Toulouse, Toulouse, France) and Volker Müller (Department of Molecular Microbiology & Bioenergetics, Institute of Molecular Biosciences, Goethe-University Frankfurt am Main, Frankfurt am Main, Germany), respectively, as they are not publicly available. The genomes of strains DSM 105863 and SA11 were publicly available and obtained from the NCBI database.

**Table 1:** *Eubacterium* strains used in this study

Current strain designation of strains according to DSMZ	Strain designation	Collection numbers	Accession number	Genome size [bp]	G + C content [mol%]	CDS	Genes	rRNAs; tRNAs	Illumina coverage	Illumina mapping rate	Nanopore coverage	Nanopore mapping rate	References
*Eubacterium limosum*	Type strain	DSM 20543; ATCC 8486; NCIB 9763	CP019962.1	4,422,837	47.2	4,090	4,209	16; 61	–	–	–	–	[12]
*Eubacterium limosum*	B2	not publicly available	CP097376.1	4,421,327	47.2	4,085	4,205	16; 51	–	–	–	–	[20]
*Eubacterium callanderi*	FD, type strain	DSM 3662; ATCC 49165; JCM 10284	JAVVAB000000000.1	4,369,831	47.3	4,033	4,109	16; 59	189x	96.66%	259x	97.59%	This study
*“Butyribacterium methylotrophicum”*	Marburg	DSM 3468; ATCC 33266	CP132155.1 CP132156.1	4,278,751	47.5	3,979	4,075	16; 61	93x	99.75%	331x	99.53%	This study
*Eubacterium callanderi*	KIST612	not publicly available	CP002273.2	4,316,707	47.5	4,052	4,158	16; 58	–	–	–	–	[23]
*“Eubacterium limosum”*	2A	DSM 2593; JCM 10283	CP132135.1	4,612,903	46.9	4,269	4,385	16; 60	214x	99.83%	232x	99.85%	This study
*“Eubacterium limosum”*	11A	DSM 2594	CP132136.1	4,612,907	46.9	4,291	4,406	16; 60	152x	99.9%	206x	98.92%	this study
*“Eubacterium limosum”*	G14	DSM 107592	CP132137.1	4,409,437	47.5	4,051	4,181	16; 61	163x	98.09%	174x	97.7%	this study
*Eubacterium maltosivorans*	YI, type strain	DSM 105863; JCM 32297	CP029487.1	4,337,501	47.8	4,005	4,118	16; 65	–	–	–	–	[71]
*“Eubacterium sp.”*	32	DSM 20517; ATCC 10825; NCIB 9554; NCTC 10469	CP132138.1	4,152,794	47.6	3,861	3,958	16; 60	178x	99.83%	337x	99.76%	this study
*“Eubacterium limosum”*	SA11	not publicly available	CP011914.1	4,150,332	47.4	3,805	3,922	16; 60	–	–	–	–	[81]

Strain designations written with quotation marks were reassigned according to dDDH, AAI, and ANIm analysis (see Fig. 3A and Additional File 1: Fig. S6)

- Not applicable

Strains purchased from DSMZ were grown from lyophilized stock cultures in 5 mL DSM 104 medium and cultivated anaerobically in hungate tubes at 37 °C. Afterwards, cells of respective strains were cultivated using modified DSM 135 medium as described before, which contained 200 mM methanol and 60 mM KHCO_3_ as carbon source [19]. Heterotrophic batch experiments were conducted in triplicates in 50 mL modified DSM 135 medium in 125-mL Müller-Krempel flasks supplemented with 250 mM methanol and N_2_:CO_2_ (80:20) in the headspace pressurized to 1 bar. For cloning purposes, chemically competent *E. coli* DH5α cells were cultivated in liquid (whilst shaking) or on solid lysogenic broth (1% tryptone, 1% NaCl, 0.5% yeast extract) supplemented with 250 μg mL^−1^ erythromycin and cultivated at 37 °C. Recombinant *Eubacterium* strains were cultivated in modified DSM 135 medium supplemented with 20 mM glucose in presence of 5 µg mL^−1^ clarithromycin.

### Analytics

Cell culture supernatants were analyzed using the PerkinElmer Clarus 680 GC system (Perkin Elmer LAS GmbH, Waltham, MA, USA) gas-chromatograph equipped with an Elite-FFAP capillary column (length 30 m x inner diameter 0.32 mm, film thickness 0.25 μm) (Perkin Elmer LAS GmbH, Waltham, MA, USA) and FID detector. Supernatants were acidified using 2 M HCl. H_2_ was used as the carrier gas. The injector and detector were operated at 225 and 300 °C, respectively. 1 μL of supernatant was injected and analyzed regarding acetate, butyrate, hexanoate, isobutyrate, isovalerate, methanol, and valerate using the following temperature profile: 40 °C for 2.5 min; 40 °C to 250 °C with 30 °C min^−1^; 250 °C for 2 min.

### Illumina and nanopore sequencing

High molecular weight DNA (HWD) was isolated with the MasterPure Complete DNA & RNA Purification kit as recommended by the manufacturer (Biozym, Hessisch Oldendorf, Germany). Quality of isolated DNA was initially checked by agarose gel electrophoresis and validated using an Agilent Bioanalyzer 2100 and the Agilent DNA 12000 kit as recommended by the manufacturer (Agilent Technologies, Waldbronn, Germany). Concentration and purity of the isolated DNA was first checked with a Nanodrop ND-1000 (PeqLab, Erlangen, Germany) and concentration was determined using the Qubit® dsDNA HS Assay kit as recommended by the manufacturer (Life Technologies GmbH, Darmstadt, Germany). Illumina paired-end libraries were prepared using the Nextera XT DNA Sample Preparation kit. To assess quality and size of the libraries, samples were analyzed employing a Agilent Bioanalyzer 2100 and Agilent High Sensitivity DNA kit as recommended by the manufacturer (Agilent Technologies, Waldbronn, Germany). Concentration of the libraries were determined using the Qubit® dsDNA HS Assay Kit as recommended by the manufacturer (Life Technologies GmbH, Darmstadt, Germany). Sequencing was performed using a MiSeq system and the reagent kit v3 with 600 cycles as recommended by the manufacturer (Illumina, San Diego, CA, USA). For Nanopore sequencing, 1.5 µg HWD were used for library preparation using the Ligation Sequencing kit 1D (SQK-LSK109) and the Native Barcode Expansion kit (EXP-NBD104 and EXP-NBD114) as recommended by the manufacturer (Oxford Nanopore Technologies, Oxford, United Kingdom). Sequencing was performed for 72 h using a MinION device Mk1B and a SpotON Flow Cell R9.4.1 according to the manufacturer (Oxford Nanopore Technologies, Oxford, United Kingdom).

### Bioinformatics

MinKNOW software version 21.10.4 was employed for sequencing and Guppy version 6.0.1 in high accuracy mode for basecalling and demultiplexing. Illumina short reads were quality filtered using Trimmomatic (v0.39; LEADING: 3, TRAILING: 3, SLIDINGWINDOW:4:15, MINLEN:50) [32]. Nanopore long reads were first quality filtered with fastp (v0.23.2) [33] and then adapter trimmed using porechop (v0.2.4). Unicycler version 0.4.8 was used with default settings to perform a hybrid assembly. Genome assemblies were inspected by Bandage [34] and BRIG to validate the GC-skew to detect any potential missassemblies [35]. Annotation was performed with Prokka and default settings [36]. Circular maps were prepared using the BLAST Ring Image Generator (BRIG) [35] using blastn [37] for genome comparison. Genomic Islands were determined using IslandViewer4 [38] and phage regions with PHASTEST [39]. The phylogeny of the strains was analyzed by multilocus sequence analysis (MLSA). Clusters of orthologous groups were identified using proteinortho version 6.031 [40], in-paralogs removed, sequences aligned using MUSCLE [41], and poorly aligned positions automatically filtered from the alignments using Gblocks [42]. The maximum-likelihood tree from 2341 orthologous groups was inferred with 500 bootstraps with RAxML version 8.1.22 [43]. The script PO_2_MLSA.py is available at github (https://github.com/jvollme). The tree was visualized using Dendroscope version 3.5.9 [44]. ANIm values were calculated using the JSpeciesWS webtool [45]. Average amino acid identities (AAI) analysis and AAI-distance clustering was performed using the AAI matrix calculator [46]. Digital DNA-DNA hybridization (dDDH) was performed using the Genome-to-Genome Distance Calculator (GGDC 3.0) [47, 48].

### Data availability statement

Genome sequence of *Eubacterium callanderi* DSM 2593 was deposited under BioProject accession PRJNA1001321, the assembly under accession number CP132135.1, and the raw sequence data under SRR27198268 (Illumina) and SRR27198267 (Nanopore). Genome sequence of *Eubacterium callanderi* DSM 2594 was deposited under BioProject accession PRJNA1001323, the assembly under accession number CP132136.1, and the raw sequence data under SRR27198387 (Illumina) and SRR27198386 (Nanopore). Genome sequence of *Eubacterium callanderi* DSM 107592 was deposited under BioProject accession PRJNA1001322, the assembly under accession number CP132137.1, and the raw sequence data under SRR27198385 (Illumina) and SRR27198384 (Nanopore). Genome sequence of *Eubacterium maltosivorans* DSM 20517 was deposited under BioProject accession PRJNA1001326, the assembly under accession number CP132138.1, and the raw sequence data under SRR27198272 (Illumina) and SRR27198271 (Nanopore). Genome sequence of *Eubacterium callanderi* DSM 3662^ T^ was deposited under BioProject accession PRJNA1001325, the assembly under accession number VVIL00000000.1, and the raw sequence data under SRR27198270 (Illumina) and SRR27198269 (Nanopore). Genome sequence of *Eubacterium callanderi* DSM 3468 was deposited under BioProject accession PRJNA1001324, the assembly under accession number CP132155.1 (chromosome) and CP132156.1 (plasmid), and the raw sequence data under SRR27198274 (Illumina) and SRR27198273 (Nanopore).

### Plasmid construction

Plasmids and primers used in this study are listed in Tables 2 and 3. DNA fragments were amplified using “KAPA Hifi “ (Kapa Biosystem, Sigma-Aldrich Chemie GmbH, Munich, Germany) or “CloneAmp” (Takara Bio, Saint-Germain-en-Laye, France) polymerase. Primers were ordered at biomers.net GmbH (Ulm, Germany). DNA used for cloning purpose was purified by gel extraction using the “NucleoSpin Gel and PCR clean-up kit” (Macherey–Nagel GmbH & Co. KG, Düren, Germany). For construction of plasmid pMTL83251_P_*fd*__FAST, the promoter region of pMTL83251_P_*bgaL*__FAST was exchanged with the ferredoxin promoter of *C. ljungdahlii*. Therefore, P_*fd*_ was amplified from genomic DNA of *C. ljungdahlii* using primers FW_Pfd_CLJU_NdeI and RV_Pfd_CLJU_BamHI and assembled with pMTL83251_P_*bgaL*__FAST digested using restriction enzymes *Nde*I and *Bam*HI to excise P_*bgaL*_. Fragments were assembled using the “NEBuilder Hifi DNA Assembly Cloning Kit” according to the manufacturer’s protocol (New England Biolabs, Ipswich, MA, USA). Plasmid pMTL82251_P_*bgaL*__FAST was constructed by simultaneous PCR amplification of *feg* and the promoter P_*bgaL*_ from plasmid pMTL83251_P_*bgaL*__FAST using primers FW_PbgaL_NdeI and RV_Pthlsup_FAST_XhoI. Fragments were assembled with *Nde*I- and *Xho*I-digested pMTL82251 plasmid DNA. Plasmid pMTL82251_P_*fd*__FAST was constructed by amplifying the fragment containing the promoter P_*fd*_ and *feg* from pMTL83251_P_*fd*__FAST using primers FW_Pfd_CLJU_NdeI as well as RV_Pthlsup_FAST_XhoI. Fragments were assembled with *Nde*I- and *Xho*I-digested pMTL82251 plasmid DNA. The construction of plasmids pJIR751_P_*bgaL*__FAST and pJIR751_P_*fd*__FAST was performed by restriction digestion of pJIR751 using *Bam*HI and subsequent assembly of PCR-amplified promoter-FAST fragments. Therefore, fragments containing P_*bgaL*_ and *feg* or P_*fd*_ and *feg* were amplified using primers FW_PbgaL-FAST-terminator as well as RV_PbgaL-FAST-terminator from plasmids pMTL83251_P_*bgal*__FAST and pMTL83251_P_*fd*__FAST, respectively.

**Table 2:** Primers used in this study

Primer	Sequence 5′–3′	Length [bp]
FW_Pfd_CLJU_NdeI	gaccgcggccgctgtatccatatgtcactatctgcggaacctg	43
RV_Pfd_CLJU_BamHI	gaccgcggccgctgtatccatatgtcactatctgcggaacctg	47
FW_PbgaL_NdeI	gaccgcggccgctgtatccatatgtaatttagatattaattctaaattaagtgaaattaatatag	65
RV_Pthlsup_FAST_XhoI	aagcttgcatgtctgcaggcctcgagtcataccctcttaac	41
FW_PbgaL-FAST-terminator	aattcgagctcggtacccggataaaaaaattgtagataaattttataaaatagttttatc	60
RV_PbgaL-FAST-terminator	gcctgcaggtcgactctagaataaaaataagaagcctgcaaatg	44

**Table 3:** Plasmids used in this study

Plasmid	Description	Source
pMTL83251_P_*bgaL*__FAST	ColE1 ori^−^, pCB102 ori^+^, Em^r^, *traJ*, *lacZ*, *bgaR-*P_*bgaL*_ from *C. perfringens, feg*	[19]
pMTL83251_P_*fd*__FAST	ColE1 ori^−^, pCB102 ori^+^, Em^r^, *traJ*, *lacZ*, P_*fd*_ from *C. ljungdahlii, feg*	This work
pMTL82251	ColE1 ori^−^, pBP1 ori^+^, Em^r^, *traJ*, *lacZ*	[49]
pMTL82251_P_*bgaL*__FAST	pMTL82251, *bgaR-*P_*bgaL*_ from *C. perfringens, feg*	This work
pMTL82251_P_*fd*__FAST	pMTL82251, P_*fd*_ from *C. ljungdahlii, feg*	This work
pJIR751	pMB1 ori^−^, pIP404 ori^+^, Em^r^	(50)
pJIR751_P_*bgaL*__FAST	pJIR751, *bgaR-*P_*bgaL*_ from *C. perfringens, feg*	This work
pJIR751_P_*fd*__FAST	pJIR751, P_*fd*_ from *C. ljungdahlii, feg*	This work

### Transformation

*Eubacterium* strains were transformed as described before [19]. In brief, cells of strains DSM 20543^T^, B2, DSM 3662^T^, DSM3468, KIST612, DSM 2593, DSM 2594, DSM 107592, and DSM 20517 were cultivated overnight at 37 °C in 50 mL modified DSM 135 medium supplemented with 20 mM glucose and 40 mM DL-threonine. For the preparation of electrocompetent cells, cells were harvested, washed two times with anaerobic SMP buffer (270 mM sucrose, 1 mM MgCl_2_, 7 mM NaH_2_PO_4_, pH 6) (7.690 × g for 10 min at 4 °C), suspended in 648 µL SMP and 72 µL DMSO under strictly anaerobic conditions in an anaerobic chamber (gas atmosphere 95% N_2_ and 5% H_2_). 3–5 µg plasmid DNA were added to 25 µL of electrocompetent cells and transferred into a pre-cooled 1 mm electroporation cuvette (Biozym Scientific GmbH, Oldendorf, Germany). Cells were pulsed (625 V, 25 μF, 600 Ω; Gene Pulser Xcell™, Bio-Rad Laboratories GmbH, Munich, Germany), transferred into 5 mL fresh DSM 135 medium supplemented with 20 mM glucose, and recovered at 37 °C. After 1–2 doublings of cells, 5 µg mL^−1^ clarithromycin was added and cells further incubated. Cells growing in presence of clarithromycin were transferred into 5 mL fresh modified DSM 135 medium supplemented with 20 mM glucose and 5 µg mL^−1^ clarithromycin. Successful transformation of cells was confirmed by determining fluorescence using flow cytometry followed by plasmid isolation using the Zyppy™ Plasmid Miniprep Kit (Zymo Research, Irvine, CA, USA), retransformation of respective plasmid DNA in *E. coli* cells, and subsequent analytical digestion of isolated plasmid DNA.

### Fluorescence determination

Fluorescence of recombinant cells of respective strains was determined using the SYNERGY H1 microplate reader (BioTek, Bad Friedrichshall, Germany) and the Amnis® CellStream® flow cytometer (Luminex Corporation, Austin, TX, USA). Initially, cells were harvested and washed with cold PBS (137 mM NaCl, 2.7 mM KCl, 10 mM Na_2_HPO_4_, 1.8 mM KH_2_PO_4_; 7711 × g, 10 min, 4 °C), afterwards recovered in cold PBS, and adjusted to an OD of 1. The microplate reader assay was set up by first transferring 100 µL of cell suspension of OD 1 to black flatbottomed 96-well microtiter plates (Greiner Bio-One GmbH, Frickenhausen, Germany) and supplementing with 5 μM ^TF^Lime (The Twinkle Factory, France, Paris). Cells were excited at 480 nm and emission determined at 541 nm. Both wavelengths correspond to the maxima of the fluorogen ^TF^Lime. For flow cytometry, cells adjusted to OD 1 were diluted 1:100 in pre-cooled PBS, transferred to round bottomed 96-well plates, and supplemented with 5 μM ^TF^Lime. 10,000 events were recorded, and fluorescence was assessed at an excitation wavelength of 488 nm using a 528/46 nm emission filter. Acquired flow cytometry data were analyzed using the CellStream™ Analysis tool version 1.2.152 (Luminex Corporation, Austin, TX, USA).

## Results

### Genomic features and phylogenetic analysis of *Eubacterium* sp.

**Fig. 1:**
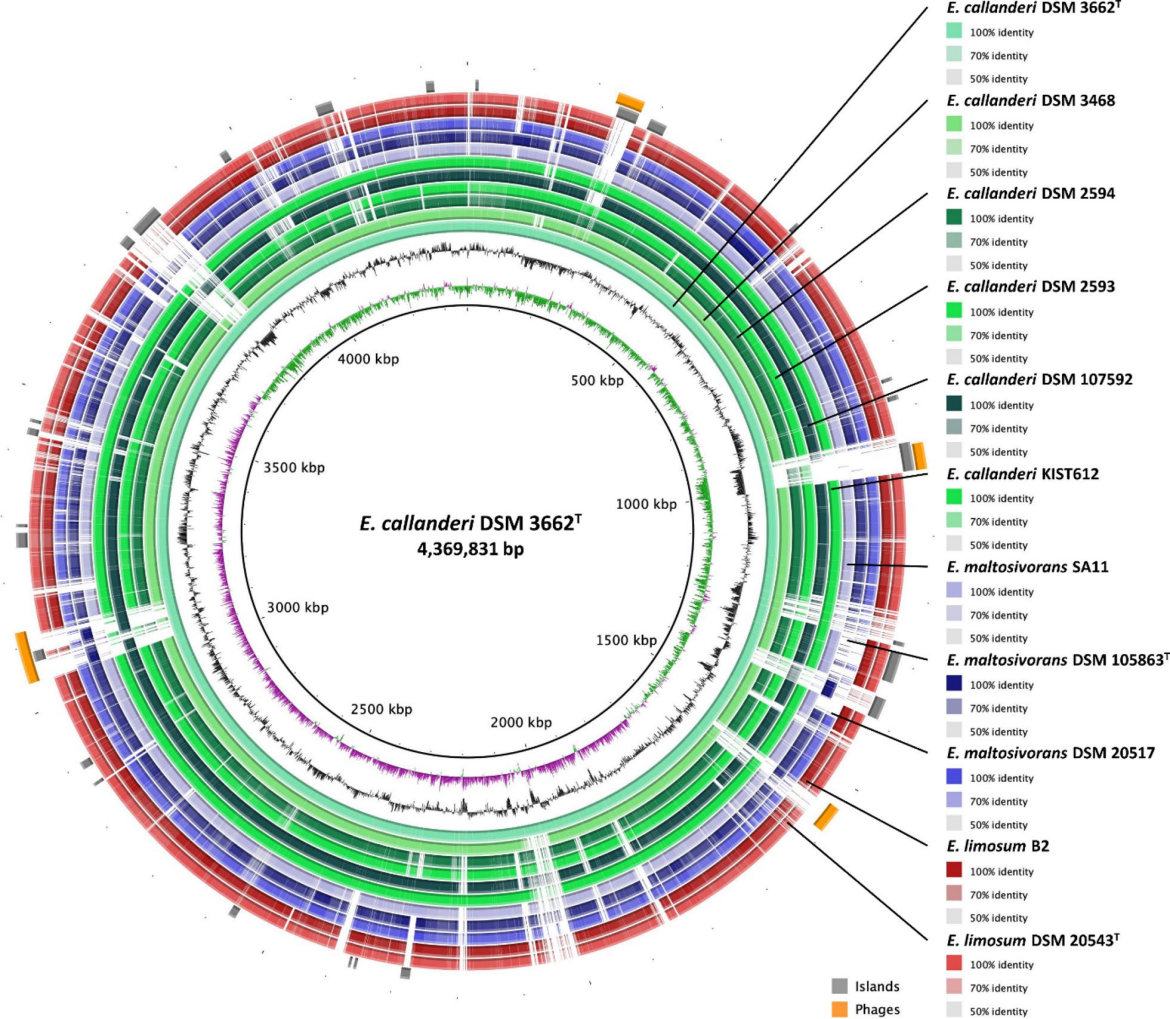
Whole genome comparison of *E. callanderi* DSM 3662^T^ with closely related *Eubacterium* strains. For a better and easier visualization of a circular map we concatenated both contigs of *E. callanderi* DSM 3662^T^ into one artificial scaffold. The reference genome of *E. callanderi* DSM 3662^T^ and its size is indicated by the inner circle. The second and third circle represent the GC skew and GC content, respectively. *E. callanderi* strains are displayed in green, *E. maltosivorans* strains in purple, and *E. limosum* strains in red nuances. Orthologous genes are indicated with high, medium, and low identity showcased by respective color gradient in the figure legend. Phage regions (orange) and GIs (grey) are displayed on the outer circles

**Fig. 2:**
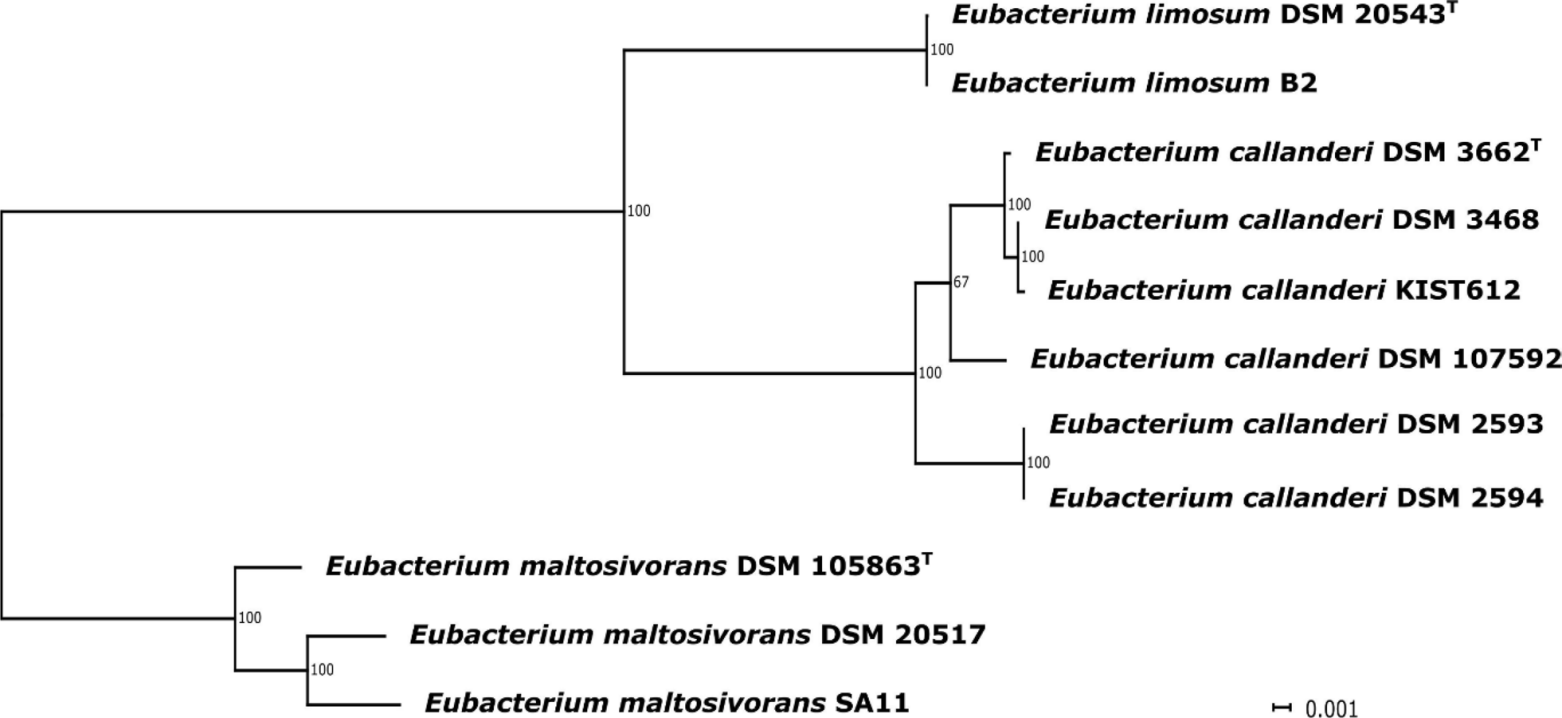
MLSA maximum-likelihood phylogenetic tree of the analyzed *Eubacterium* strains. The alignment was created from 2341 orthologous groups present in all genomes. The tree was inferred with 500 bootstraps with RAxML. The lengths of the tree branches were scaled according to the number of substitutions per site

A whole genome comparison was performed with the eleven *Eubacterium* strains either obtained from the database of the National Center for Biotechnology Information (NCBI) or by de novo sequencing in this study. (Table 1; Fig. 1, Additional File 1: Figs. S1–S5). Current strain designations according to DSMZ, culture collection numbers, genome accession numbers, genome size, and other relevant information of the eleven *Eubacterium* strains are listed in Table 1. The genomes of all listed strains consist of one circular chromosome of 4.2–4.4 Mb and an overall G + C content of 47–48 mol% (Table 1). Only strain ‘Marburg’ (DSM 3468) harbors a potential plasmid (CP132156.1) with the size of 39,785 bp and a G + C content of 45%. Respective plasmid only encodes hypothetical proteins. Biochemical evidence for the presence of this plasmid is missing so far. Interestingly the genomes of strain ‘2A’ (DSM 2593) and ‘11A’ (DSM 2594) are nearly identical and only differ by four nucleotides (Table 1, Additional File 1: Figs. S2–S3). Both presence of genomic islands (GIs) and phage-associated genes were detected in the sequenced genomes of strains ‘FD’ (DSM 3662^T^), ‘Marburg’ (DSM3468), ‘2A’ (DSM 2593), ‘11A’ (DSM 2594), ‘G14’ (DSM 107592), and ‘32’ (DSM 20517). Phage-associated gene clusters were spread throughout the genomes ranging from two to five regions (Fig. 1, Additional File 1: Figs. S1–S5; Additional File 2). Various GIs were identified in the six sequenced strains, which mainly encode hypothetical proteins (Fig. 1, Additional File 1: Figs. S1–S5; Additional File 3). While only 19 GIs were identified in strain ‘32’ (DSM 20517) (Additional File 1: Fig. S5), 34 GIs were identified in the genome of strains ‘2A’ (DSM 2593) and ‘11A’ (DSM 2594) (Additional File 1: Figs. S1–S2). MLSA was used to generate a phylogenetic tree based on the identified core genome of 2341 OGs, which yielded three distinct clades (Fig. 2).

Clade one comprises the type strain of *E. limosum* (DSM 20543^T^) and strain ‘B2’, clade two the type strain of *E. callanderi* ‘FD’ (DSM 3662^T^) as well as the strains ‘Marburg’ (DSM 3468), ‘KIST612’, ‘2A’ (DSM 2593), ‘11A’ (DSM 2594), and G14 (DSM 107592), and the third clade the type strain of *E. maltosivorans* (DSM 105863^T^), strain ‘SA11’, and ‘32’ (DSM 20517).

**Fig. 3:**
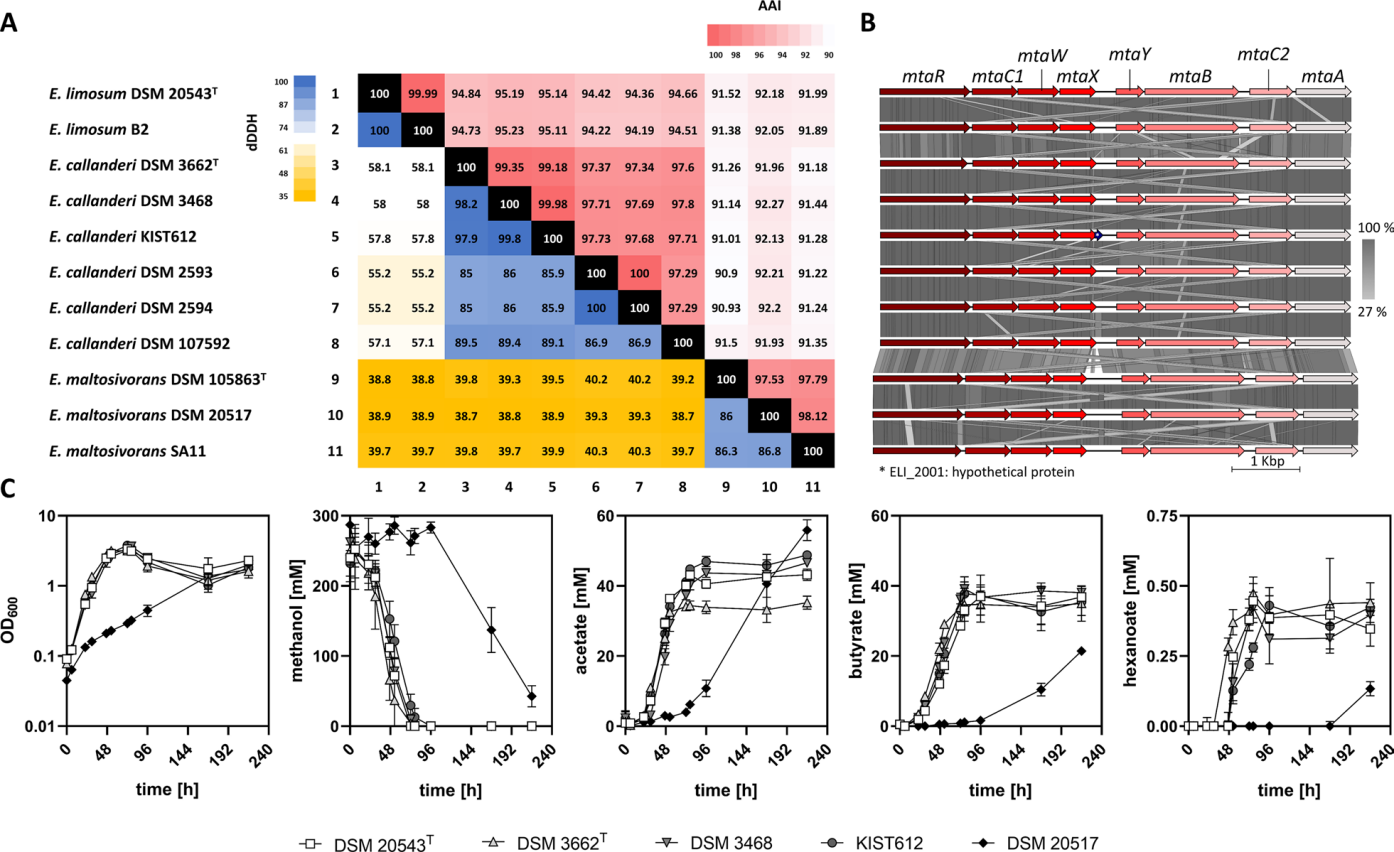
Phylogenetic, genomic, and physiological comparison of methanol-utilizing *Eubacterium* strains. **A** Pairwise digital DNA-DNA hybridization values and average amino acid identities of the eleven *Eubacterium* strains. Strains are separated into three distinct clades comprising *E. limosum*, *E. callanderi*, and *E. maltosivorans*. **B** Arrangement of the methyltransferase system genes in the analyzed *Eubacterium* strains. **C** Growth behavior of methanol-utilizing *Eubacterium* strains represented by at least one strain of each clade (*E. limosum* clade; type strain DSM 20543^T^, *E. callanderi* clade; type strain DSM3662.^T^, strain ‘Marburg’ DSM 3468, and ‘KIST612’, as well as the *E. maltosivorans* clade; ‘32’ DSM 20517). From left to right, optical density, methanol utilization, acetate-, butyrate-, and hexanoate production. Error bars indicate standard deviations. n = 3

Species boundaries among the strains were further analyzed by determining digital DNA-DNA hybridization (dDDH) values, average amino acid identities (AAI), and average nucleotide identities based on the MUMmer algorithm (ANIm) (Fig. 3A, Additional File 1: Fig. S6). Again, three clades were identified based on the pairwise dDDH, AAI, and ANIm values.

The type strain of *E. limosum* DSM 20543^T^ and strain ‘B2’ represented the first clade with identical dDDH values. The second clade comprised the type strain of *E. callanderi* DSM 3662^T^ with dDDH values above 70% when compared to those of the strains DSM 3468 (98.2%), ‘KIST612’ (97.9%), DSM 2593 (85%), DSM 2594 (85%), and DSM 107592 (89.5%). The third clade comprised the type strain *E. maltosivorans* DSM 105863^T^ that shared high dDDH identities with those of strains ‘SA11’ (86.3%), and DSM 20517 (86%) (Fig. 3A).

AAI and ANIm analysis matches the results of dDDH, resulting in the same three distinct clades (Fig. 3A). The type strain of *E. limosum* DSM 20543^T^ and ‘B2’ share 99.9% AAI identity and about 95% or 92% when compared to strains of the other two clades. The type strain of *E. callanderi* DSM 3662^T^ shares high AAI with the strains DSM 3468 (99.35%), ‘KIST612’ (99.18%), DSM 2593 (97.37%), DSM 2594 (97.34%), and DSM 107592 (97.6%). AAI values revealed 99.89% identity between the strains DSM 3468 and ‘KIST612’. The AAI of strains DSM 2593 and DSM 2594 were identical. The third clade comprised the type strain *E. maltosivorans* DSM 105863^T^, which shares 97.79% and 97.53% identities with strain ‘SA11’ and DSM 20517, respectively.

Based on the ANIm matrix (Additional File 1: Fig. S6) the first clade comprised the type strain of *E. limosum* DSM 20543^T^ and strain ‘B2’_,_ showing an ANIm value of 99.9%. The second clade comprised the type strain of *E. callanderi* DSM 3662^T^, which shared high identities when compared to genomic data of the strains DSM 3468 (99.79%), ‘KIST612’ (99.77%), DSM 2593 (98.42%), DSM 2594 (98.42%), and DSM 107592 (98.91%). The third clade comprised the type strain *E. maltosivorans* (DSM 105863^T^), as well as strains ‘SA11’, and DSM 20517.

### *Eubacterium* strains perform chain elongation from methanol

Acetogens utilize methanol via the Wood-Ljungdahl pathway, which requires a functional methyltransferase system [51]. This enzyme complex is composed of the subunits methyltransferase I (*mtaB*), corrinoid protein (*mtaC2*), and methyltransferase II (*mtaA*). The genomes of all analyzed strains harbor the respective operons and their gene arrangement is similar to that of the operon encoding the methyltransferase system of *A. woodii* [52] (Fig. 3B). Moreover, analyzed *Eubacterium* strains were able to utilize methanol as shown by respective growth experiments (Fig. 3C). The representative strains of the *E. limosum* clade (DSM 20543^T^) and the *E. callanderi* clade (DSM 3662^T^, DSM 3468, and KIST612) utilized methanol with a consumption rate of 4.896–6.551 mM h^−1^ and showed growth rates of about 0.07 h^−1^ (Fig. 3C; Table 4). Strain ‘32’ (DSM 20517), a representative of the *E. maltosivorans* clade, consumed methanol after an initial lag phase with a consumption rate of only 2.005 mM h^−1^ and showed a rather poor growth rate of 0.02 h^−1^ (Fig. 3C; Table 4). All these five strains produced acetate and butyrate as main metabolic products, while also traces of hexanoate were detected. Hexanoate is produced via chain elongation as described in detail for *Clostridium* *kluyveri* or *Clostridium carboxidivorans* [53, 54]*.* Therefore, the genes *thl, crt*, *hbd*, and *bcd* (encoding crotonase, 3-hydroxybutyryl-CoA dehydrogenase, thiolase, and butyryl-CoA dehydrogenase, respectively) coupled to the genes encoding electron-transferring flavoproteins *etfA* and *etfB* forming the *hcs* operon are necessary. All genomes of the analyzed *Eubacterium* strains harbor genes of the *bcs*/*hcs* operon in the same arrangement (Additional File 1: Fig. S7). Acetate and butyrate production rates during exponential growth were similar for the type strain of *E. limosum* (DSM 20543^T^), the type strain of *E. callanderi* (DSM 3662^T^), as well as the strains ‘Marburg’ (DSM 3468) and ‘KIST612’ with 0.914–1.271 mM acetate h^−1^ and 0.646–0.870 mM butyrate h^−1^, respectively (Fig. 3C; Table 4). Strain ‘32’ (DSM20517) produced both products with production rates of 0.379- and 0.162-mM h^−1^. Hexanoate was produced with production rates ranging from 0.002- to 0.011 mM h^−1^ indicating the potential of chain elongation (Fig. 3C; Table 4). We could not detect any ethanol or butanol production in the performed growth experiments. However, genome analysis revealed several potential genes, which gene products could be involved in alcohol production in the strains of all three clades. The genome of all strains encodes at least one potential aldehyde:ferredoxin oxidoreductase and alcohol dehydrogenase. For instance, based on annotations, the genome of the type strain of *E. limosum* (DSM 20543^T^) encodes three alcohol dehydrogenases (B2M23_09455, B2M23_12405, B2M23_15170) and a butanol dehydrogenase (B2M23_02350), which might convert butyraldehyde to butanol and two aldehyde:ferredoxin oxidoreductases (B2M23_16025, B2M23_04655), potentially converting butyrate to butyraldehyde. Same genes are annotated in strain B2; three alcohol dehydrogenases (M5595_07965, M5595_18545, M5595_00635), a butanol dehydrogenase (M5595_15090,) and two aldehyde:ferredoxin oxidoreductases (M5595_01490, M5595_12770).

**Table 4:** Growth, consumption, and production rates of *Eubacterium* strains cultivated using methanol

Strain	Growth rate [h^−1^]	Methanol consumption rate [mM h^−1^]	Acetate production rate [mM h^−1^]	Butyrate production rate [mM h^−1^]	Hexanoate production rate [mM h^−1^]
*Eubacterium limosum* DSM 20543^T^	0.067	6.058	1.271	0.646	0.011
*Eubacterium callanderi* DSM 3662^T^	0.063	6.551	0.914	0.870	0.006
*Eubacterium callanderi* DSM 3468	0.067	6.099	1.018	0.750	0.011
*Eubacterium callanderi* KIST612	0.067	4.896	1.088	0.703	0.007
*Eubacterium* maltosivorans DSM 20517	0.020	2.005	0.379	0.162	0.002

The *E. callanderi* clade includes the natural butanol producing strains ‘KIST612’ and ‘Marburg’ (DSM 3468). The genome of strain KIST 612 encodes at least four potential alcohol dehydrogenases (and ELI_0942, ELI_2986, ELI_0037, ELI4403) and one potential *aor* gene (ELI_3389). Identical gene sequences are present in the genome of strain ‘Marburg’ (DSM 3468) (EUCAMar_37310, EUCAMar_01710, EUCAMar_27100, EUCAMar_29180, EUCAMar_18280). The type strain of *E. callanderi* (DSM 3662^T^) is not described to produce butanol, but at least four alcohol dehydrogenase are annotated (EUCAFD_23750, EUCAFD_27570, EUCAFD_37320, EUCAFD_40080).

Finally, it should be mentioned that microscopic examinations provided no indication that cells of any *Eubacterium* strain sporulate during the performed growth experiments. This result was confirmed by genome analysis, which revealed that the sporulation specific gene *spo0A* is not present in the analyzed strains. However, several other genes are annotated that are potentially related to sporulation, but the respective phenotype was never observed in the performed experiments.

### *Eubacterium* strains are genetically accessible

**Fig. 4:**
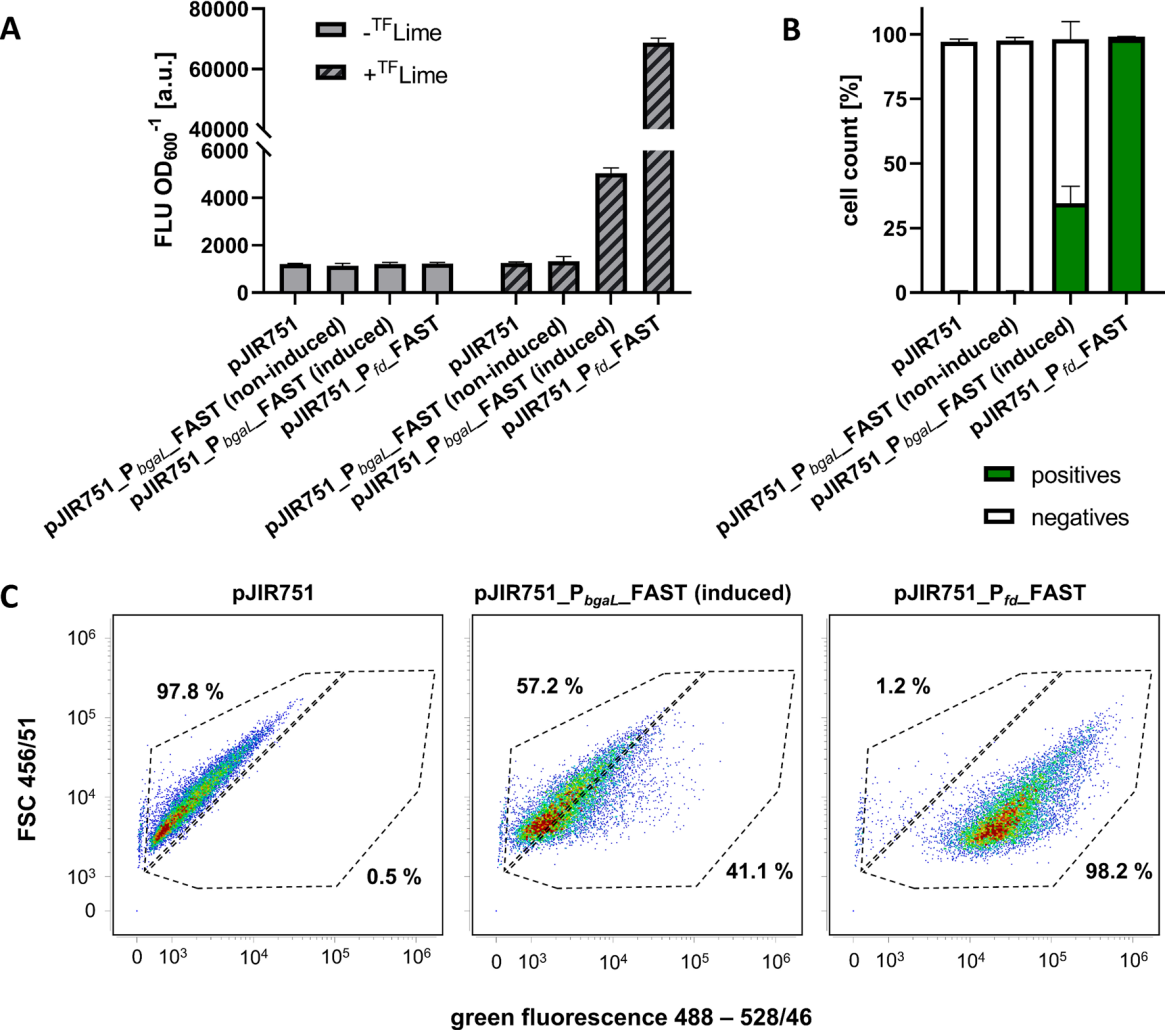
Improved recombinant FAST production with strain ‘B2’ resulting in a homogeneous fluorescent cell population. **A** Fluorescent intensity of the whole cell population of recombinant ‘B2’ strains determined using a microplate reader in presence or absence of the fluorogen ^TF^Lime. Lactose-induced *feg* expression controlled by P_*bgaL*_ caused weak and constitutive expression controlled by P_*fd*_ strong fluorescence. No fluorescence was determined in absence of.^TF^Lime, by the empty vector control, or by non-induced cells. **B** Number of fluorescent recombinant ‘B2’ cells determined using flow cytometry. **C** Density plots of recombinant ‘B2’ strains. All ‘B2’cells harboring the empty vector control pJIR751 were non-fluorescent. Lactose-induced expression of *feg* caused an overall weak fluorescent heterogeneous population. Expression of *feg* controlled by the constitutive P_*fd*_ promoter resulted in a homogeneous, brightly fluorescent population of recombinant ‘B2’ [pJIR751_Pfd_FAST] cells. Error bars indicate standard deviations. n = 3

Four of the evaluated *Eubacterium* strains have already been described to be genetically accessible, namely the type strain of *E. limosum* (DSM 20543^T^), ‘B2’, ‘Marburg’ (DSM 3468), and ‘KIST612’. In addition, we investigated the genetic accessibility of five more strains namely the type strain of *E. callanderi* (DSM 3662^T^), ‘2A’ (DSM 2593), ‘11A’ (DSM 2594), ‘G14’ (DSM 107592), and ‘32’ (DSM 20517) in this study. Previously, we reported the use of FAST as reporter protein in strain ‘B2’ [19, 55]. Strain ‘B2’ showed bright fluorescence, whereby heterogeneous populations of fluorescent and non-fluorescent cells were detected. However, to use FAST as a reporter for high-throughput and real-time screenings, a homogenous fluorescent cell population was desired. Thus, we improved our previously established FAST reporter system, which was based on the lactose-inducible *bgaR*-P_*bgaL*_ promoter system of *C. perfringens*. Hence, strain ‘B2’ [pJIR751_P_*fd*__FAST] was constructed, which expressed the FAST-encoding gene (*feg*) under control of the strong constitutive ferredoxin promoter (P_*fd*_) from *Clostridium ljungdahlii* (Fig. 4A). Fluorescence intensity of the entire cell population of ‘B2’ [pJIR751_P_*fd*__FAST] was determined using a microplate reader and compared to fluorescence of cells derived from the strains ‘B2’ [pJIR751_P_*bgaL*__FAST] and ‘B2’ [pJIR751]. The fluorescence intensity of ‘B2’ [pJIR751_P_*fd*__FAST] cells was 13.6-fold improved when compared to ‘B2’ [pJIR_P_*bgaL*__FAST]. Moreover, a 54.7- and 51.9-fold increase in fluorescence intensity was determined when compared to non-fluorescent cells of the empty vector control ‘B2’ [pJIR751] as well as the non-induced cells of ‘B2’ [pMTL83251_P_*bgaL*__FAST], respectively (Fig. 4A). Furthermore, the number of fluorescent cells was determined at a single-cell level using flow cytometry (Fig. 4B). 98.2% of ‘B2’ [pJIR751_P_*fd*__FAST] cells were green fluorescent resulting in a homogenous population (Fig. 4B, C). Compared to that, the empty vector control strain ‘B2’ [pMTL83251] caused a homogenous population of non-fluorescent cells, while the population of ‘B2’ [pMTL83251_P_*bgaL*__FAST] was still heterogeneous and only caused poor fluorescence (Fig. 4B, C).

**Fig. 5:**
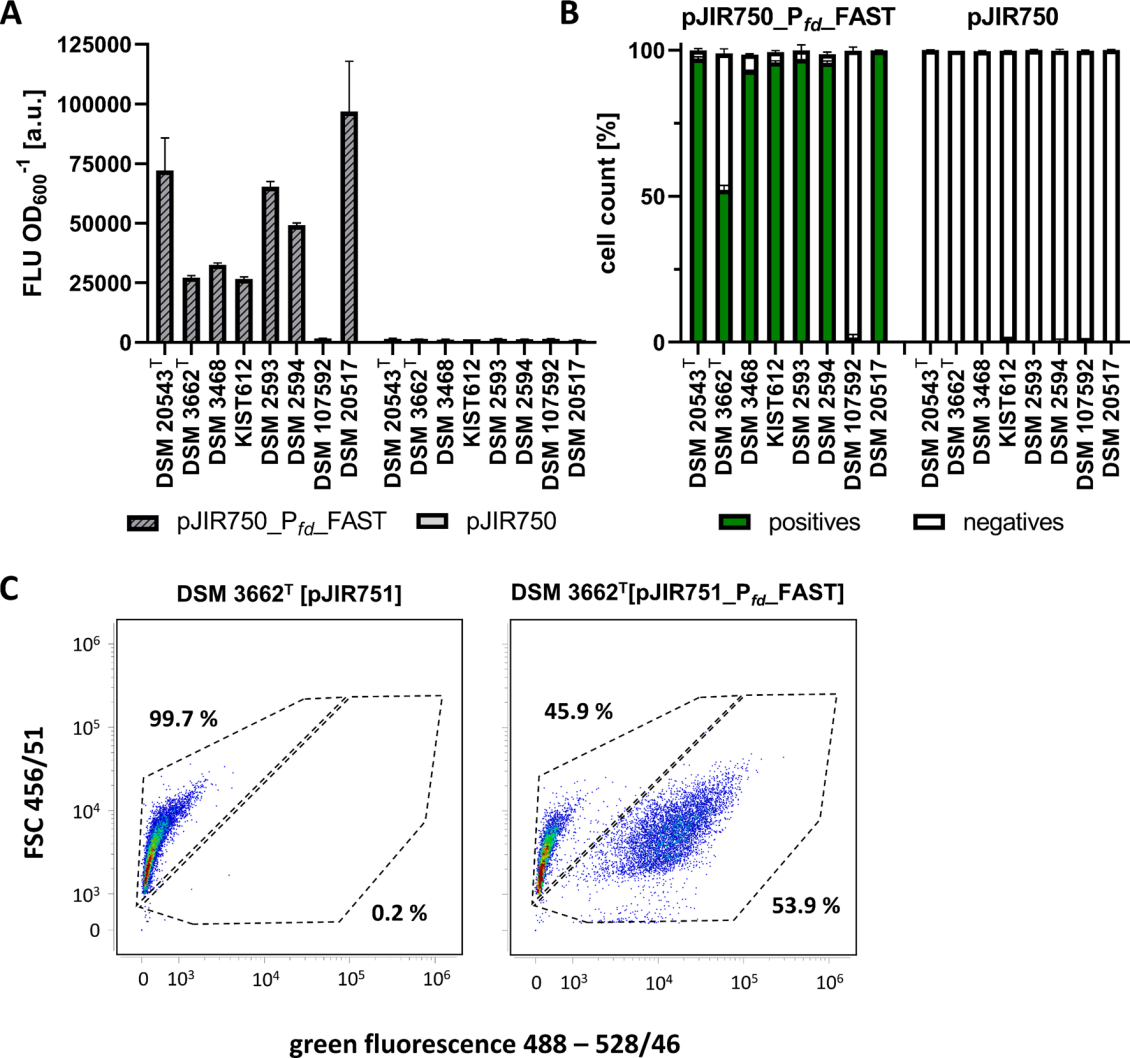
Verification of genetic accessibility of the type strains of *E. limosum* (DSM 20543^T^) and *E. callanderi* ‘FD’ (DSM 3662^T^), as well as the strains ‘Marburg’ (DSM3468), ‘KIST612’, ‘2A’ (DSM 2593), ‘11A’ (DSM 2594), ‘G14’ (DSM 107592), and ‘32’ (DSM 20517) based on the FAST-mediated fluorescence. **A** Fluorescence intensities of the whole cell populations of recombinant strains were determined using a microplate reader in presence of the fluorogen ^TF^Lime. Successfully transformed strains resulted in bright fluorescence. Strains harboring the empty vector control were non-fluorescent. Strain ‘G14’ was not transformed and was therefore non-fluorescent. **B** Number of fluorescent cells of the recombinant strains determined using flow cytometry. **C** Density plots of recombinant DSM 3662^T^ [pJIR751] and DSM 3662T [pJIR751_P_*fd*__FAST] cells. Expression of *feg* resulted in a heterogeneous population of DSM 3662.^T^ [pJIR751_P_*fd*__FAST]. Error bars indicate standard deviations. n = 3

This P_*fd*_ controlled *feg* expression system was used for rapid screening of successfully constructed recombinant cells of the type strain of *E. limosum* (DSM 20543^T^), the type strain of *E. callanderi* (DSM 3662^T^), as well as the strains ‘Marburg’ (DSM3468), ‘KIST612’, ‘2A’ (DSM 2593), ‘11A’ (DSM 2594), ‘G14’ (DSM 107592), and ‘32’ (DSM 20517). Therefore, respective cells were electroporated using the plasmid pJIR751_P_*fd*__FAST and the empty vector control pJIR751. All strains that were transformed with these plasmids showed growth in the presence of clarithromycin. Fluorescence of respective cells was determined using the microplate reader after being transferred and regrown in fresh medium supplemented with clarithromycin to verify successful transformation. The recombinant cells of the type strains of *E. limosum* (DSM 20543^T^), and of *E. callanderi* (DSM 3662^T^), as well as the strains ‘Marburg’ (DSM3468), ‘KIST612’, ‘2A’ (DSM 2593), ‘11A’ (DSM 2594), and ‘32’ (DSM 20517) showed bright fluorescence, while ‘G14’ (DSM 107592) was non-fluorescent (Fig. 5A). In more detail, recombinant FAST-producing strain ‘32’ (DSM 20517) [pJIR751_P_*fd*__FAST] representing the *E. maltosivorans* clade showed the highest overall fluorescence (Fig. 5A). The fluorescence of *E. limosum* (DSM 20543^T^) [pJIR751_P_*fd*__FAST] was in the same range as fluorescence of strain ‘B2’ [pJIR751_P_*fd*__FAST], both being members of the *E. limosum* clade (Figs. 4A and 5A). The lowest fluorescence signals were determined for recombinant strains of the *E. callanderi* clade, namely the type strain of *E. callanderi* (DSM 3662^T^) [pJIR751_P_*fd*__FAST], as well as strains ‘Marburg’ (DSM 3468) [pJIR751_P_*fd*__FAST] and ‘KIST612’ [pJIR751_P_*fd*__FAST] (Fig. 5A). However, recombinant cells of FAST-producing strains ‘2A’ (DSM 2593) and ‘11A’ (DSM 2594) resulted in fluorescence in the range of strains of the *E. limosum* clade (Fig. 5A). Only weak autofluorescence was determined for strains harboring the empty vector control (Fig. 5A). In addition, the number of fluorescent cells was determined using flow cytometry (Fig. 5B). FAST production of recombinant cells of the type strain of *E. limosum* (DSM 20543^T^), the strains ‘Marburg’ (DSM3468), ‘KIST612’, ‘2A’ (DSM 2593), ‘11A’ (DSM 2594), and ‘32’ (DSM 20517) resulted in homogenous populations with 93–99.8% fluorescent cells. However, flow cytometry data revealed that recombinant cells of the type strain of *E. callanderi* (DSM 3662^T^) [pJIR751_P_*fd*__FAST] caused a heterogeneous population consisting of 52.1% positive- and 46.7% negative cells (Fig. 5B, C). As shown before, cells of strain ‘G14’ (DSM 107592) were non-fluorescent, which resulted in a homogenous non-fluorescent population. Afterwards, plasmids were verified by isolation, retransformation in *E. coli* cells, and analytical digestion of reisolated plasmid DNA. No plasmid could be isolated from cells of strain ‘G14’ (DSM 107592), matching previous results.

Moreover, plasmids pMTL82251 and pMTL82251_P_*fd*__FAST were constructed. Strains electroporated with respective plasmids showed no growth in the presence of clarithromycin.

## Discussion

### Reclassification of *Eubacterium* strains

Three distinct clades comprising the eleven analyzed *Eubacterium* strains were classified based on the phylogenetic analysis, dDDH-, ANIm-, and AAI values. We concluded that the strains of respective clades belong to the same species and consequently propose to reclassify the strains ‘Marburg’ (DSM 3468), ‘2A’ (DSM 2593), ‘11A’ (DSM 2594), ‘G14’ (DSM 107592), ‘32’ (DSM 20517), and ‘SA11’ as follows.

### Genomic and physiological variations in *Eubacterium limosum*

Based on our data, the only two strains belonging to the species *E. limosum* are the type strain (DSM 20543^T^) and strain ‘B2’, together forming the *E. limosum* clade. Pregnon and co-workers recently compared the genomes of both strains in detail and identified only 21 differences including single nucleotide polymorphisms as well as single nucleotide insertions and deletions [20]. Physiologically, these two strains differ regarding the formation of extracellular polymeric substances (EPS), which are largely produced by the *E. limosum* type strain (DSM 20543^T^) but not by the ‘B2’ strain. Recently, the genes encoding gene products mediating the EPS formation were identified and deleted in DSM 20543^T^ [8]. However, the respective wild type genomic region is identically present in strain ‘B2’ making its lack of EPS formation still puzzling. More importantly, our results disproved the proclamation that *E. limosum* and strain ‘32’ (DSM 20517) (formerly known as *Butyribacterium rettgeri*) are “synonyms”, since strain ‘32’ clearly belongs to the *E. maltosivorans* clade [17, 18].

### Emended description of *Eubacterium limosum* Eggerth 1935, Prévot 1938 (Approved Lists 1980), emend. Cato et al. 1981

A detailed description of the species *E. limosum* is summarized and given by Wade [56]. The genome of *E. limosum* contains genes for the WLP, genes of the methyltransferase system, and of the *bcs*/*hcs* operon. Metabolic products are acetate, butyrate, and hexanoate. *E. limosum* produces traces of butanol from the co-substrates methanol and formate [7]. The type strain as well as strain ‘B2’ are both genetically accessible [9, 11, 19]. Strain ‘32’ (DSM 20517), formerly known as ‘*Butyribacterium rettgeri’*, does not belong to the species *E. limosum*. So far, the type strain (DSM 20543^T^ = ATCC 8486^T^ = NCIB 9763^T^) and strain ‘B2’ are the only strains belonging to the species *E. limosum*. Strain ‘RF’ is not sequenced and not publicly available in any strain collection and was therefore not considered for this study.

### The species *Eubacterium callanderi* is diverse and comprises potent C1 utilizers

Based on the phylogenetic analysis, dDDH-, ANIm-, and AAI values the type strain of *E. callanderi* (DSM 3662^T^), strains ‘Marburg’ (DSM 3468), ‘KIST612’, ‘2A’ (DSM 2593), ‘11A’ (DSM 2594), and ‘G14’ (DSM 107592) form the *E. callanderi* clade. Although the initial description of the type strain of *E. callanderi* (DSM 3662^T^) shows similarities to *E. limosum*, some differences were highlighted, which justified the new species description [25, 26]. Major differences mentioned for *E. callanderi* in separation from *E. limosum* were the inability of cells to utilize one-carbon substrates, cells need acetate in defined medium to grow on glucose, and cells produce acetate, butyrate, formate, lactate, and H_2_. In this study, we showed that *E. callanderi* (DSM 3662^T^) cells utilize methanol and also harbor the required MTI-encoding gene cluster. Moreover, strains of the *E. callanderi* clade were cultivated with glucose in the absence of acetate during this study. However, we could not detect any formate production in our experiments. Transient formate production is described for various acetogens. *Acetobacterium woodii* and *Eubacterium* strains are capable of converting this C1 carbon source via the WLP [7, 57, 58]. The same is true for lactate and H_2_ [15]. Differences in substrate utilization and product spectrum might be due to different cultivation conditions, therefore we avoid making any substantiated statements regarding formate, lactate, and H_2_ production for these strains. Furthermore, just as all other strains of the *E. limosum* and *E. callanderi* clade, *E. callanderi* ‘FD’ (DSM 3662^T^) harbors all genes of the *bcs*/*hcs* operon and produces hexanoate via chain elongation. Although originally described as *E. limosum*, strain ‘KIST612’ was recently assigned as *E. callanderi* ‘KIST612’ [21, 24, 59]. Correspondingly, our experiments and data clearly show that strain ‘KIST612’ is a member of the *E. callanderi* species*.* Moreover, the strain ‘Marburg’ (DSM 3468), which was initially assigned to the genus ‘*Butyribacterium’*, clusters with the type strain of *E. callanderi* (DSM 3662^T^). Their genome sequences are highly similar with a dDDH similarity of 98.2 and AAI similarity of 99.35%. Both values exceed the threshold for distinct species of 70 and 95% based on dDDH and AAI similarities, respectively. Therefore, strain ‘Marburg’ (DSM 3468) (also known as ‘*B. methylotrophicum*’) needs to be renamed into *E. callanderi*, which was already contemplated in the past [27]. Although strain ‘Marburg’ (DSM 3468) seemed to differ from the type strain of *E. limosum* (DSM 20543^T^), an accurate comparison of both strains was never performed [60]. Initially, strain ‘Marburg’ (DSM 3468) was described to form atypical spores, which distinguishes cells from those of the genus *Eubacterium* and was used to justify the genus ‘*Butyribacterium’* [28]. We could not confirm sporulation of cells and only identified four genes in the genome of strain ‘Marburg’ (DSM 3468), which are associated regarding their annotation with sporulation (EUCAMar_24330, EUCAMar_28780, EUCAMar_28790, and EUCAMar_30560). Those genes are also annotated in the genomes of all sequenced *Eubacterium* strains, which are all non-spore formers. Moreover, strain ‘Marburg’ (DSM 3468) and ‘KIST612’ share 99.8% dDDH similarity, while AAI shows 99.98% identity. This finding indicates that strains ‘Marburg’ and ‘KIST612’ are identical and could be treated synonymously. The same is true for strains ‘2A’ (DSM 2593) and ‘11A’ (DSM 2594) as their dDDH and AAI are identical. The potential of strain’Marburg’ (DSM 3468) to produce butanol from CO was exceptional for quite some time [29, 61]. Later on, butanol production was reported for the type strain of *E. limosum* (DSM20543^T^) and for ‘KIST612’ [7, 62]. In general, alcohol formation by acetogens is quite common and well elucidated for *C. ljungdahlii* and *C. autoethanogenum* and meanwhile even commercialized by the company LanzaTech [63]. However, both clostridial strains lack genes of the *bcs* operon and, therefore, butanol production was just achieved in recombinant strains [64, 65]. So far, the only other acetogen that naturally and reproducibly produces considerable amounts of butanol and hexanol is *C. carboxidivorans* [66, 67]. Alcohol formation in acetogens mostly depends on alcohol dehydrogenases and aldehyde:ferredoxin oxidoreductases, which are present in the genome of all *Eubacterium* strains analyzed and facilitate the possibility of native butanol production. To prove the capability of butanol production on a physiological level, the amount of available reducing equivalents has to be improved during cultivation [16]. Therefore, experiments with CO or high methanol concentrations compared to the co-substrates CO_2,_ HCO_3_^−^, or formate have to be performed [7, 61]. Besides improving alcohol formation, increasing the methanol/co-substrate ratio can also enhance hexanoate production via β-oxidation, which seems to be the preferred metabolic route in *E. limosum* [68].

We could not identify any physiological difference between strains of the *E. limosum* and *E. callanderi* clade. Strains of the two clades share high AAI and ANIm similarities close to the threshold of 95% for species separation. However, based on the dDDH values, which are below the threshold of 70%, *E. limosum* and *E. callanderi* are distinct species. As a result, we assign the strains ‘Marburg’ (DSM3468), ‘KIST612’, ‘2A’ (DSM 2593), ‘11A’ (DSM 2594), and ‘G14’ (DSM 107592) as members of the species *E. callanderi*. Based on our results and those described in the literature for the ‘Marburg strain’ and ‘KIST612’ the description of *E. callanderi* is emended as written below.

### Emended description of *Eubacterium callanderi* Mountfort et al. 1988

The description is given by Mountfort et al. (Mountfort et al. 1988) with the following modifications. Cells of *E. callanderi* grow with glucose as the sole carbon source. Cells utilize C1 carbon sources including methanol, formate, CO, and H_2_ + CO_2_. In addition to acetate, butyrate, lactate, and H_2_ *E. callanderi* produces hexanoate from methanol and butanol from CO. The genome of *E. callanderi* contains all genes of the WLP, genes of the methyltransferase system, and of the *bcs*/*hcs* operon. The strains *E. callanderi* ‘FD’ (DSM 3662^T^), ‘Marburg’ (DSM 3468), ‘KIST612’, ‘2A’ (DSM 2593), and ‘11A’ (DSM 2594) are genetically accessible. These insights were gained among others by genome sequencing, transformation, and growth experiments with the *E. callanderi* strains ‘FD’ (DSM 3662^T^), ‘KIST612’, ‘2A’ (DSM 2593), ‘11A’ (DSM 2594), and ‘G14’ (DSM 107592) using media and performing analytics as described in the materials and methods section as well as results described in the literature [29, 69, 70]. Strain ‘Marburg’ formerly known as ‘*Butyribacterium methylotrophicum*’, is reclassified as *Eubacterium callanderi* ‘Marburg’ (DSM 3468 = ATCC 33266). Strains ‘KIST612’, ‘2A’, ‘11A’, and ‘G14’, formerly described as *E. limosum*, are reclassified to *Eubacterium callanderi* ‘KIST612’, *Eubacterium callanderi* ‘2A’ (DSM 2593), *Eubacterium callanderi* ‘11A’ (DSM 2594), and *Eubacterium callanderi* ‘G14’ (DSM 107592), respectively.

### The strain ‘*Butyribacterium rettgeri’* belongs to *Eubacterium maltosivorans*

*E. maltosivorans* DSM 105863^T^ was validly described and represents the type strain of the *E. maltosivorans* clade [71]. Due to highly similar dDDH values of the strain ‘SA11’ (86.3%) and strain ‘32’ (DSM 20517) (86%) compared to the type strain DSM 105863^T^, both strains belong to the species *E. maltosivorans*. Although strain ‘32’ (DSM 20517; formerly known as ‘*Butyribacterium rettgeri’*) was isolated before *E. maltosivorans* DSM 105863^T^, it was never validly described [13, 72]. The type strain (DSM 105863^T^) was properly studied on both physiological and also phylogenetic levels and authors already proposed that strain ‘SA11’ belongs to that species [71, 73]. An extensive physiological characterization of strain ‘SA11’ was not possible since the strain is not publicly available in a public culture collection. The genome of *E. maltosivorans* (DSM 105863^T^) harbors all genes encoding the gene products mediating growth with H_2_ + CO_2_ via the WLP. Moreover, strains of the *E. maltosivorans* clade harbor genes encoding a complete methyltransferase enzyme complex highly similar to the genes of the respective operon in the *E. limosum* and *E. callanderi* strains. Although all genes are present for methanol utilization, we showed that growth of strain ‘32’ (DSM 20517) is rather weak when compared to *E. limosum* and *E. callanderi*. This finding matches the results for *E. maltosivorans* (DSM 105863^T^) by Feng and co-workers, who reported poor growth on both methanol and formate [71]. Based on 16S analysis, strain *Eubacterium* sp. ‘CS1Van’ (DSM 14465) also belongs to the *E. maltosivorans* clade. According to the literature, this strain utilizes methanol, but no other C1 carbon sources [74]. Overall, further studies on the methanol metabolism of the *E. maltosivorans* strains are necessary to give a profound reason for their weak growth on methanol and formate. We could show that the strain ‘32’ (DSM 20517) clearly differs from the type strain of *E. limosum* (DSM20543^T^) on a physiological and phylogenetic level and oppose the finding that both strains are identical and not even belong to the same species [18].

### Emended description of *Eubacterium maltosivorans* Feng et al. 2018

The description is given by Feng et al. (Feng et al. 2018) with the following modifications. The genome of *E. maltosivorans* contains genes for the WLP, genes of the methyltransferase system, and of the *bcs*/*hcs* operon. Cells of *E. maltosivorans* grow with the C1 carbon source methanol and produce acetate, butyrate, and traces of hexanoate. Strains ‘32’ and ‘SA11’ formerly described as ‘*Butyribacterium rettgeri*’ and *E. limosum*, respectively, are reclassified as *Eubacterium maltosivorans* ‘32’ (DSM 20517 = ATCC 10825) and *Eubacterium maltosivorans* ‘SA11’. Strain *E. maltosivorans* ‘32’ (DSM 20517) is genetically accessible. These insights were gained among others by genome sequencing, transformation, and growth experiments with *E. maltosivorans* ‘32’ (DSM 20517) using media and performing analytics as described in the materials and methods section.

### FAST as fluorescent reporter protein to screen for successfully transformed *Eubacterium* cells

Electroporation protocols were reported for the type strain of *E. limosum* (DSM 20543^T^), as well as strains ‘B2’, ‘Marburg’ (DSM 3468), and ‘KIST612’ [9, 11, 19, 22, 31]. The genetic toolbox includes various selection markers and promoters, plasmid-based gene expression, as well as CRISPR-Cas and CRISPRi gene editing tools. Here we successfully constructed recombinant strains of four additional strains belonging to the *E. callanderi* clade (DSM 3662^T^, DSM 2593, DSM 2594, and DSM 20517) and one belonging to the *E. maltosivorans* clade (DSM20517). By employing the fluorescent reporter protein FAST we detected fluorescent cells by applying flow cytometry. This screening method is easy, fast, and provides insights about successfully transformed cells at a single-cell level. Previously, the expression of *feg* in strain ‘B2’ caused a heterogeneous population of fluorescent and non-fluorescent cells, which indicates a limitation of this method [19]. This heterogeneity might be caused by plasmid instability, low expression levels, or insufficient induction of lactose-based (P_*bgaL*_) *feg* gene expression.

In this study, we electroporated ‘B2’ cells with plasmids harboring different origins of replications (pCB102, pIP404, and pBP1) to address the problem of plasmid instability. ‘B2’ cells could not be transformed using the plasmids harboring the origin of replication *repA* of pBP1 matching the findings described for *E. limosum* (DSM 20543^T^) (Shin et al. 2019). Shin and coworkers also showed that the transformation efficiency was low for plasmids harboring *repH* of pCB102 and high when using pIP404. By improving the electroporation protocol, *E. limosum* (DSM 20543^T^) was successfully transformed with plasmid pMTL82254 (*repA* of pBP1) proving its functionality [9]. However, when compared to plasmids with the pIP404 origin of replication, transformation efficiency was low and in the same order of magnitude as for plasmids harboring *repH* of pCB102 (pMTL83151) [9]. Interestingly, the transformation efficiency for strain ‘Marburg’ (DSM 3468) was highest when electroporating cells with plasmids harboring the ori *repH* of pCB102 and lowest with pIP404 [31]. *Eubacterium* strains in this study were successfully transformed with plasmids harboring the pIP404 origin of replication. Still, the production of FAST controlled by the lactose-inducible *bgaR*-P_*bgaL*_ system caused a heterogeneous population, regardless of which origin of replication was used.

Both, low expression levels and insufficient induction of *feg* gene expression might be caused by the inducible *bgaR*-P_*bgaL*_ promoter system. Therefore, we exchanged *bgaR*-P_*bgaL*_ with the constitutive ferredoxin promoter of *C. ljungdahlii* termed P_*fd*_. Recently, a ferredoxin promoter termed P_*fer*_ was used for strong gene expression and hence selective acetone and isopropanol production in *C. autoethanogenum* [76]. This promoter differs by an insertion of 24 bases when compared to the ferredoxin promoter (P_*fd*_) of *C. ljungdahlii* used in this study [76, 77]. Interestingly, the sequence of P_*fd*_ is identical in *C. ljungdahlii* and *C. autoethanogenum*. The strong expression of *feg* controlled by P_*fd*_ resulted in a homogenous *E. limosum* ‘B2’ [pJIR751_P_*fd*__FAST] population and caused a 13.6-fold increased fluorescence intensity compared to the respective *bgaR*_P_*bgaL*_ promoter system. This finding indicates that high expression levels can result in homogenous populations of brightly fluorescent cells, while heterogeneity might be caused by weak gene expression or insufficient induction.

We verified successfully transformed cells of strains of the *E. limosum* clade (DSM 20543^T^ and ‘B2’), the *E. callanderi* clade (‘FD’ (DSM 3662^T^), ‘Marburg’ (DSM3468), ‘KIST612’, ‘2A’ (DSM 2593), and ‘11A’ (DSM 2594)), and the *E. maltosivorans* clade (DSM 20517) using single-cell screening based on FAST mediated fluorescence. The production of FAST in respective cells resulted in homogenously fluorescent populations in seven out of the nine tested strains. Interestingly, strains that are genetically closely related and form distinct clusters in the performed AAI analysis also showed similar fluorescence intensity signals. The highest intensity was determined for strain DSM 20517 representing *E. maltosivorans*, followed by the type strain of *E. limosum* (DSM 20543^T^) and strain ‘B2’. Although *E. callanderi* ‘FD’ [pJIR751_P_*fd*__FAST] showed bright fluorescence, this strain only showed a heterogeneous population of fluorescent and non-fluorescent cells. This finding indicates that only 53.9% of cells harbor the plasmid or at least produce FAST. One limitation of recombinant acetogenic bioproduction are low titers of the desired product. Our findings suggest that heterogeneous production may also impact overall production and should be considered when constructing new production strains. In our previous studies, heterogeneity might be due to weak, lactose-induced gene expression, however, further reasons causing this phenomenon must be considered. In general, only cells harboring plasmids with a selection marker should be able to grow under antibiotic pressure. Interestingly, Sanford and Woolston speculate that low copy numbers might be caused by methylated ribosomes due to the *ermB* resistance gene, which might be transferred to daughter cells during division. Hence, cells could still be resistant to erythromycin even without harboring respective plasmid DNA [9]. This conjecture might also explain heterogeneous populations. However, it is still puzzling why this is only the case for recombinant cells of the type strain *E. callanderi* (DSM 3662^T^). Clear evidence explaining the cause of this regularly described phenomenon of heterogeneity is still missing [19, 55, 78–80].

Cells of strain ‘G14’ (DSM 107529) were not successfully transformed under any conditions tested so far. All other tested *Eubacterium* strains were capable of being transformed using the tested protocol, which opens the door for new genetically modified strains that can serve as biocatalysts for the heterologous production of biocommodities. Strains transformed with the empty vector control not harboring *feg* were verified by retransformation of *E. coli* cells with respective plasmid DNA and subsequent analytical digestion. This procedure is time-intensive and gives no insight into the number of cells harboring the respective plasmid. Due to the small size of *feg* (378 bps), FAST can easily be implemented as a fluorescent reporter to any plasmid of interest. Hence, FAST can serve as a genetic marker and can be used to rapidly screen successfully transformed cells using flow cytometry, especially for novel anaerobes that are not yet genetically accessible or suffer from low transformation efficiencies.

## Conclusion

*E. limosum*, *E. callanderi*, and *E. maltosivorans* strains are excellent candidates as biocatalysts for the anaerobic conversion of C1 substrates into valuable products. Many strains can be genetically accessed using a harmonized electroporation protocol, and FAST serves as a reliable fluorescent reporter protein for characterizing these engineered cells. A total of eleven strains are assigned to three clades, allowing a clear and up-to-date classification. Therefore, descriptions of each *Eubacterium* species were improved, adjusted, and insights should be implemented in respective official databases.

### Supplementary Information

**Additional file 1: Figure S1.** Whole genome comparison of *E. callanderi* DSM 2593 with closely related *Eubacterium* strains. The reference genome and its size is indicated by the inner circle. The second and third circle represent the GC skew and GC content, respectively. *E. callanderi *strains are displayed in green, *E. maltosivorans* strains in purple, and *E. limosum* strains in red nuances. Orthologous genes are indicated with high, medium, and low identity showcased by respective color gradient in the figure legend. Phage regions (orange) and GIs (grey) are displayed on the outer circles. **Figure S2.** Whole genome comparison of *E. callanderi* DSM 2594 with closely related *Eubacterium* strains. The reference genome and its size is indicated by the inner circle. The second and third circle represent the GC skew and GC content, respectively. *E. callanderi *strains are displayed in green, *E. maltosivorans* strains in purple, and *E. limosum* strains in red nuances. Orthologous genes are indicated with high, medium, and low identity showcased by respective color gradient in the figure legend. Phage regions (orange) and GIs (grey) are displayed on the outer circles. **Figure S3.** Whole genome comparison of *E. callanderi* DSM 3468 with closely related *Eubacterium* strains. The reference genome and its size is indicated by the inner circle. The second and third circle represent the GC skew and GC content, respectively. *E. callanderi *strains are displayed in green, *E. maltosivorans* strains in purple, and *E. limosum* strains in red nuances. Orthologous genes are indicated with high, medium, and low identity showcased by respective color gradient in the figure legend. Phage regions (orange) and GIs (grey) are displayed on the outer circles. **Figure S4.** Whole genome comparison of *E. callanderi* DSM 107592 with closely related *Eubacterium* strains. The reference genome and its size is indicated by the inner circle. The second and third circle represent the GC skew and GC content, respectively. *E. callanderi *strains are displayed in green, *E. maltosivorans* strains in purple, and *E. limosum* strains in red nuances. Orthologous genes are indicated with high, medium, and low identity showcased by respective color gradient in the figure legend. Phage regions (orange) and GIs (grey) are displayed on the outer circles. **Figure S5. **Whole genome comparison of *E. maltosivorans* DSM 107592 with closely related *Eubacterium* strains. The reference genome and its size is indicated by the inner circle. The second and third circle represent the GC skew and GC content, respectively. *E. maltosivorans* strains are displayed in purple, *E. callanderi *strains in green, and *E. limosum* strains in red nuances. Orthologous genes are indicated with high, medium, and low identity showcased by respective color gradient in the figure legend. Phage regions (orange) and GIs (grey) are displayed on the outer circles. **Figure S6. **Average nucleotide identity (ANIm) analysis of eleven *Eubacterium* strains. Strains are separated into three distinct clades comprising *E. limosum*, *E. callanderi*, and *E. maltosivorans*.** Figure S7. **Arrangement of *bcs*/*hcs* operon genes of the eleven analyzed *Eubacterium* strains.

**Additional file 2: **Phage-associated gene clusters identified in the sequenced genomes of strains ‘FD’ (DSM 3662^T^), ‘Marburg’ (DSM3468), ‘2A’ (DSM 2593), ‘11A’ (DSM 2594), ‘G14’ (DSM 107592), and ‘32’ (DSM 20517)).

**Additional file 3: **Genomic islands identified in the sequenced genomes of strains ‘FD’ (DSM 3662^T^), ‘Marburg’ (DSM3468), ‘2A’ (DSM 2593), ‘11A’ (DSM 2594), ‘G14’ (DSM 107592), and ‘32’ (DSM 20517)).

## Data Availability

All data generated during this study are included in this article and the additional files. Raw data are available on reasonable request.

## Abbreviations

AAI: Average amino acid identity
ANIm: Average nucleotide identity based on the MUMmer algorithm
EPS: Extracellular polymeric substance
FAST: Fluorescence-activating and absorption shifting tag
GIs: Genomic islands
HWD: High molecular weight DNA
MLSA: Multilocus sequence analysis
^T^: Type strain
WLP: Wood-Ljungdahl pathway
