# Supplementary material for: Refining and illuminating acetogenic Eubacterium strains for reclassification and metabolic engineering
Source: Microb Cell Fact. 2024 Jan 17;23:24. doi: 10.1186/s12934-024-02301-8 (PMC10795377; doi:10.1186/s12934-024-02301-8)
Supplement: Supplementary file 1 — Additional file 1: Figure S1. Whole genome comparison of E. callanderi DSM 2593 with closely related Eubacterium strains. The reference genome and its size is indicated by the inner circle. The second and third circle represent the GC skew and GC content, respectively. E. callanderi strains are displayed in green, E. maltosivorans strains in purple, and E. limosum strains in red nuances. Orthologous genes are indicated with high, medium, and low identity showcased by respective color gradient in the figure legend. Phage regions (orange) and GIs (grey) are displayed on the outer circles. Figure S2. Whole genome comparison of E. callanderi DSM 2594 with closely related Eubacterium strains. The reference genome and its size is indicated by the inner circle. The second and third circle represent the GC skew and GC content, respectively. E. callanderi strains are displayed in green, E. maltosivorans strains in purple, and E. limosum strains in red nuances. Orthologous genes are indicated with high, medium, and low identity showcased by respective color gradient in the figure legend. Phage regions (orange) and GIs (grey) are displayed on the outer circles. Figure S3. Whole genome comparison of E. callanderi DSM 3468 with closely related Eubacterium strains. The reference genome and its size is indicated by the inner circle. The second and third circle represent the GC skew and GC content, respectively. E. callanderi strains are displayed in green, E. maltosivorans strains in purple, and E. limosum strains in red nuances. Orthologous genes are indicated with high, medium, and low identity showcased by respective color gradient in the figure legend. Phage regions (orange) and GIs (grey) are displayed on the outer circles. Figure S4. Whole genome comparison of E. callanderi DSM 107592 with closely related Eubacterium strains. The reference genome and its size is indicated by the inner circle. The second and third circle represent the GC skew and GC content, respectively. E. [file 12934_2024_2301_MOESM1_ESM.docx]

**Additional File 1**

**
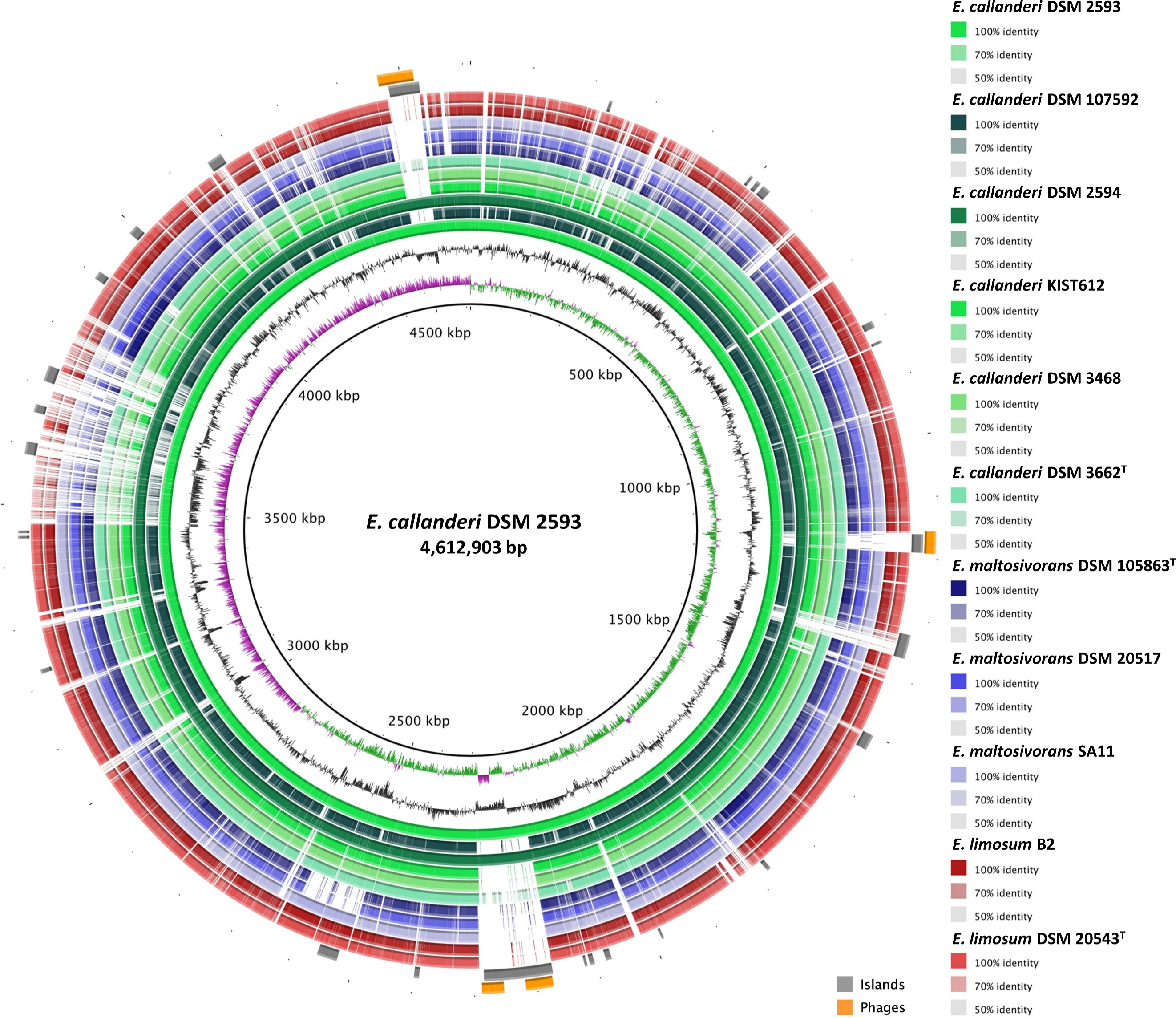
**

**Fig. S1:** Whole genome comparison of *E. callanderi* DSM 2593 with closely related *Eubacterium* strains. The reference genome and its size is indicated by the inner circle. The second and third circle represent the GC skew and GC content, respectively. *E. callanderi* strains are displayed in green, *E. maltosivorans* strains in purple, and *E. limosum* strains in red nuances. Orthologous genes are indicated with high, medium, and low identity showcased by respective color gradient in the figure legend. Phage regions (orange) and GIs (grey) are displayed on the outer circles.

**
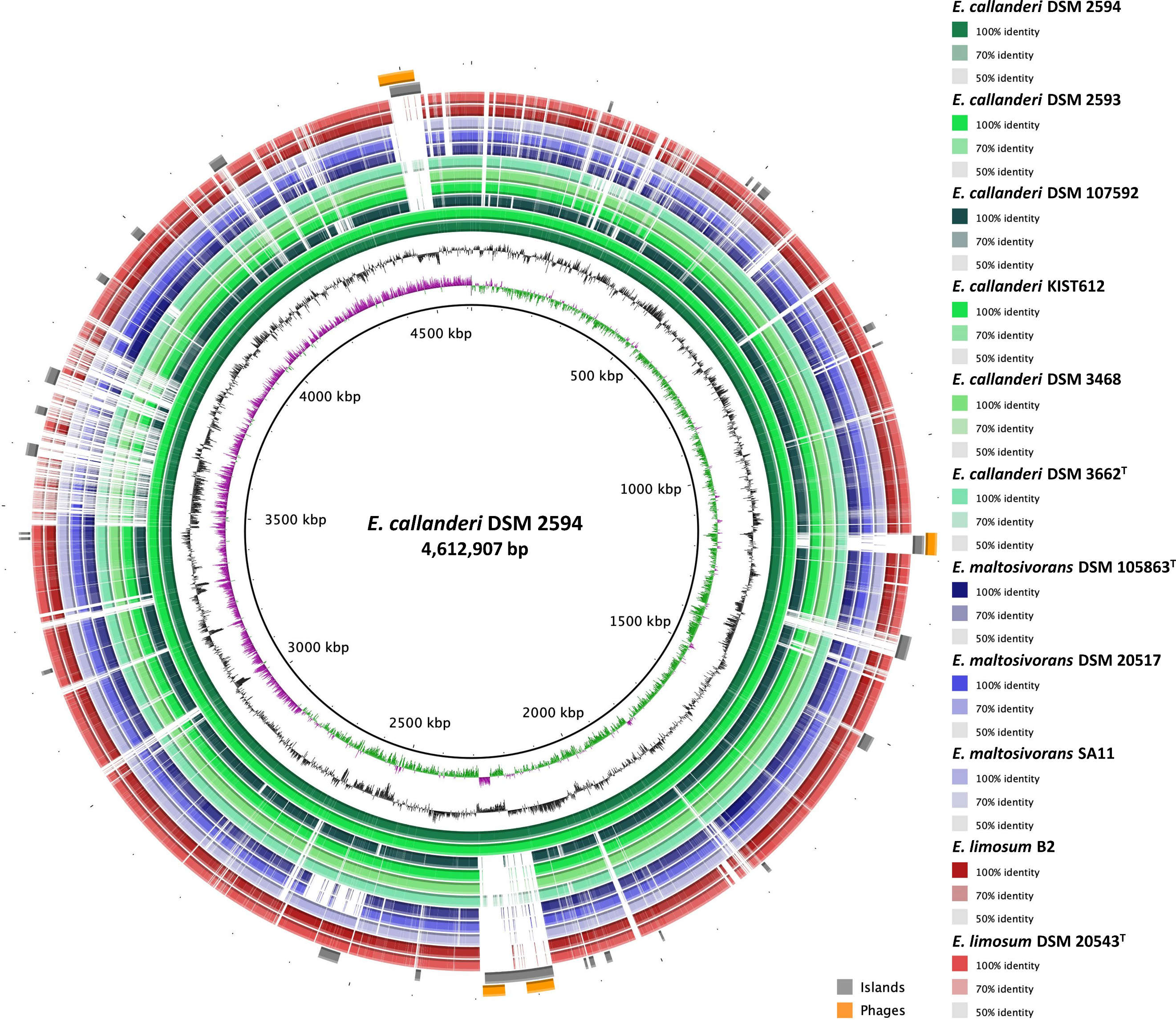
**

**Fig. S2:** Whole genome comparison of *E. callanderi* DSM 2594 with closely related *Eubacterium* strains. The reference genome and its size is indicated by the inner circle. The second and third circle represent the GC skew and GC content, respectively. *E. callanderi* strains are displayed in green, *E. maltosivorans* strains in purple, and *E. limosum* strains in red nuances. Orthologous genes are indicated with high, medium, and low identity showcased by respective color gradient in the figure legend. Phage regions (orange) and GIs (grey) are displayed on the outer circles.

**
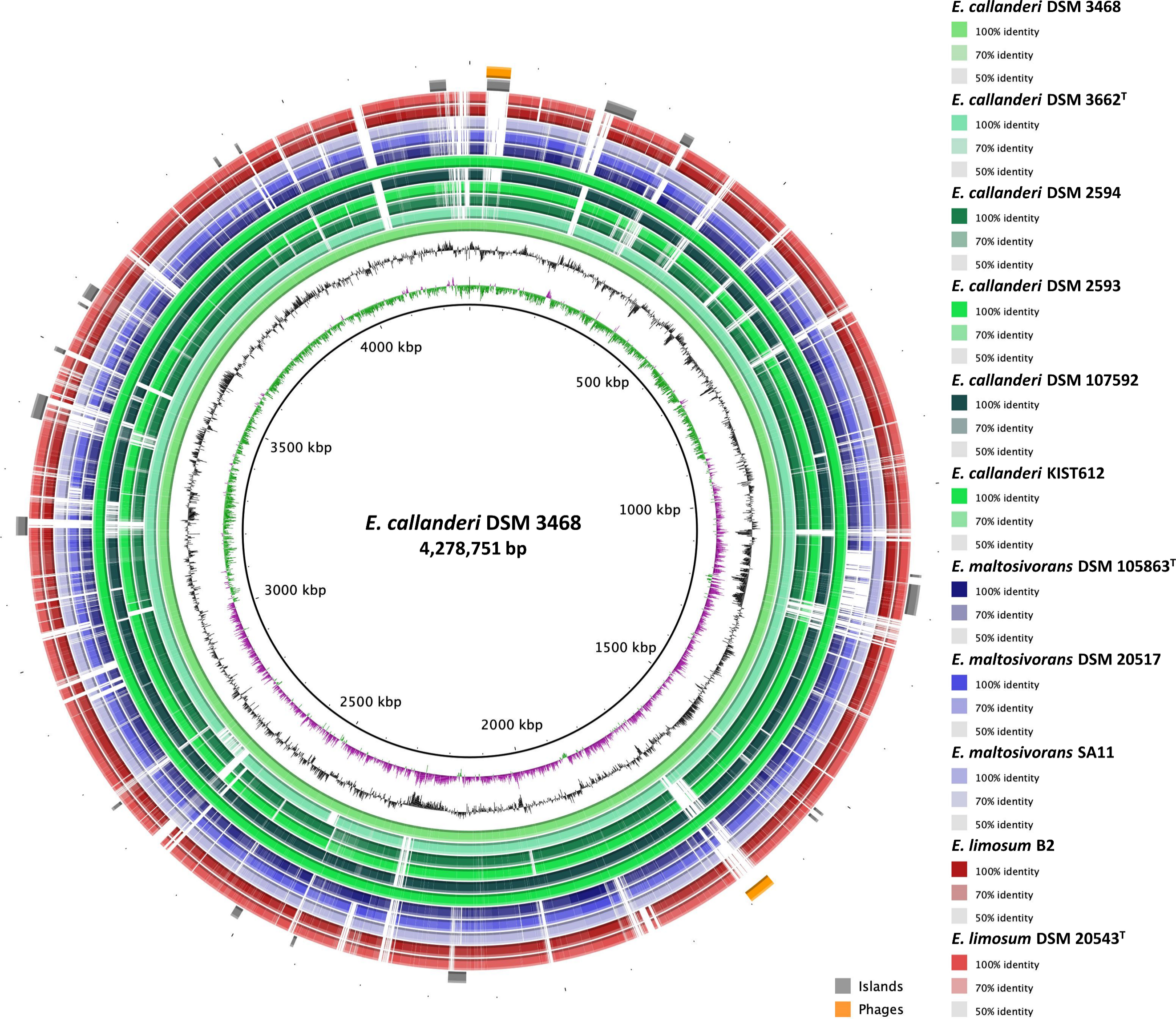
**

**Fig. S3:** Whole genome comparison of *E. callanderi* DSM 3468 with closely related *Eubacterium* strains. The reference genome and its size is indicated by the inner circle. The second and third circle represent the GC skew and GC content, respectively. *E. callanderi* strains are displayed in green, *E. maltosivorans* strains in purple, and *E. limosum* strains in red nuances. Orthologous genes are indicated with high, medium, and low identity showcased by respective color gradient in the figure legend. Phage regions (orange) and GIs (grey) are displayed on the outer circles.

**
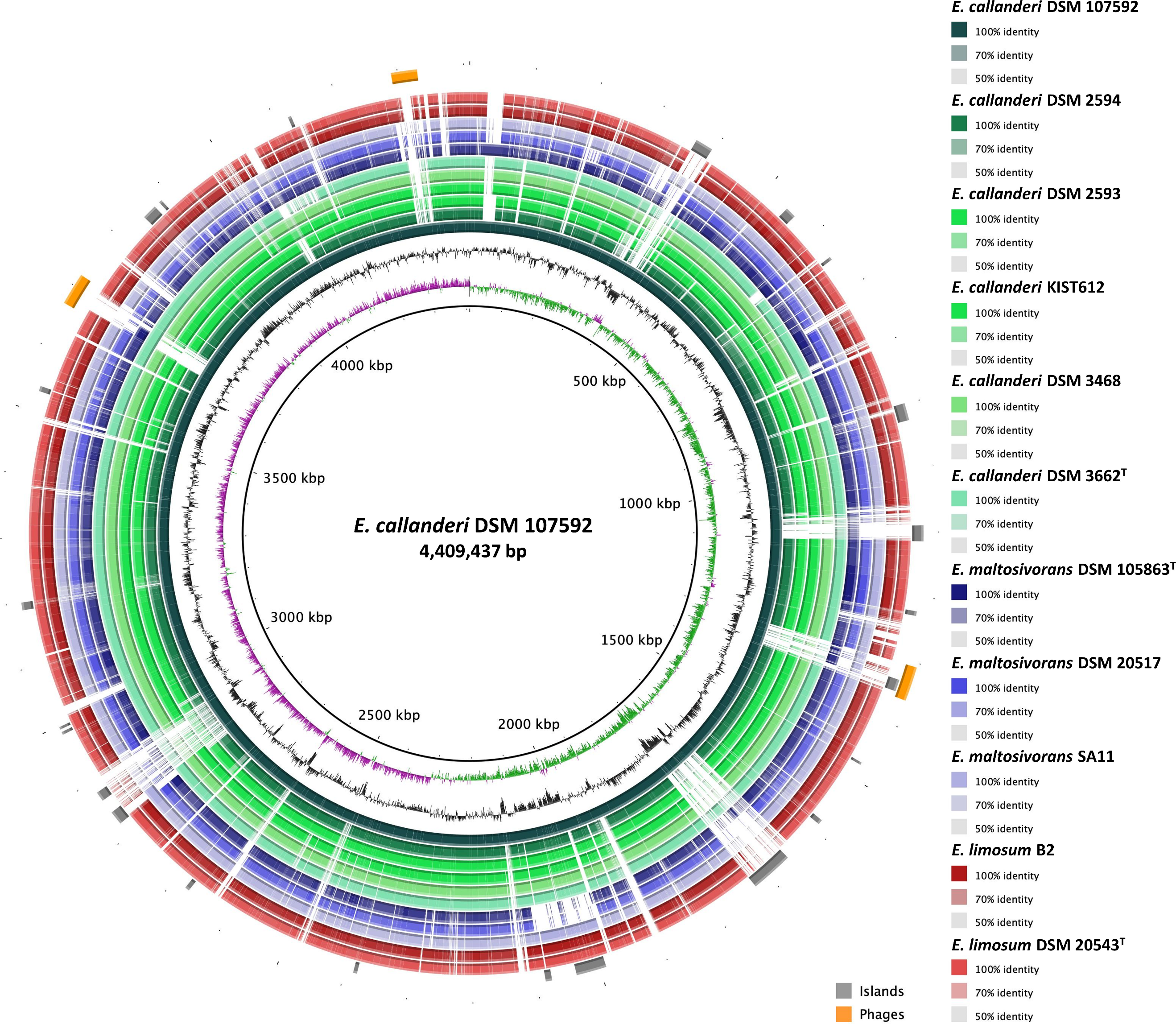
**

**Fig. S4:** Whole genome comparison of *E. callanderi* DSM 107592 with closely related *Eubacterium* strains. The reference genome and its size is indicated by the inner circle. The second and third circle represent the GC skew and GC content, respectively. *E. callanderi* strains are displayed in green, *E. maltosivorans* strains in purple, and *E. limosum* strains in red nuances. Orthologous genes are indicated with high, medium, and low identity showcased by respective color gradient in the figure legend. Phage regions (orange) and GIs (grey) are displayed on the outer circles.

**
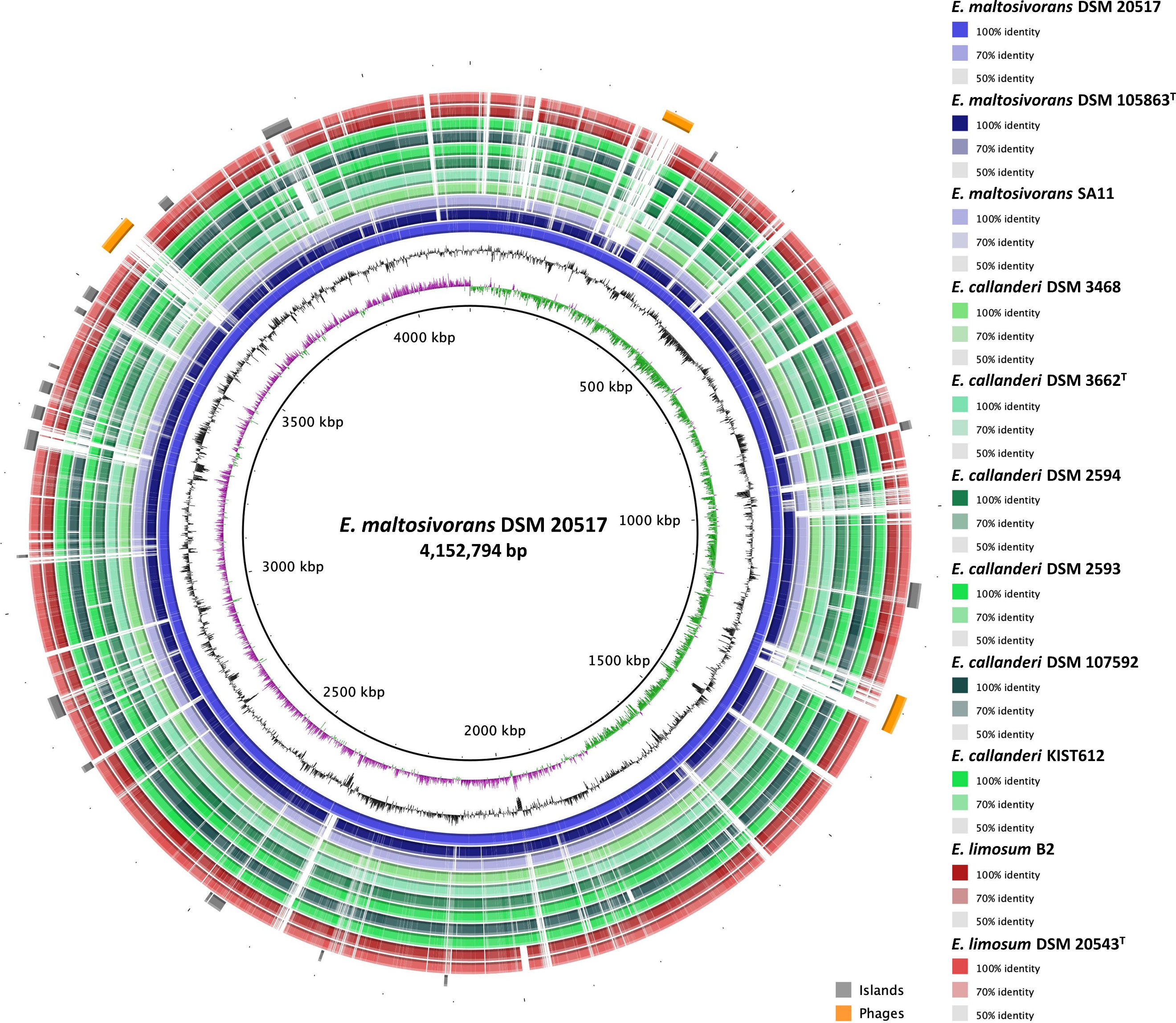
**

**Fig. S5:** Whole genome comparison of *E. maltosivorans* DSM 107592 with closely related *Eubacterium* strains. The reference genome and its size is indicated by the inner circle. The second and third circle represent the GC skew and GC content, respectively. *E. maltosivorans* strains are displayed in purple, *E. callanderi* strains in green, and *E. limosum* strains in red nuances. Orthologous genes are indicated with high, medium, and low identity showcased by respective color gradient in the figure legend. Phage regions (orange) and GIs (grey) are displayed on the outer circles.


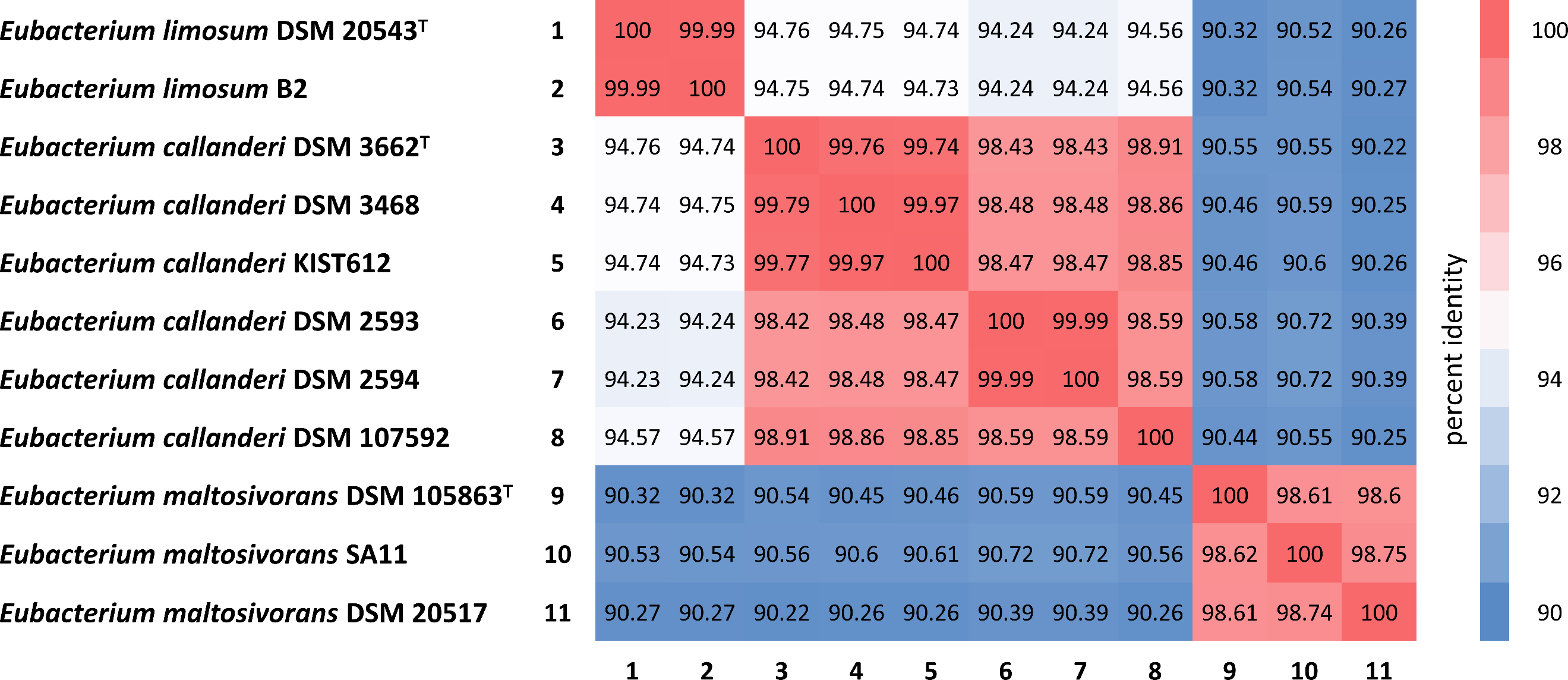
**Fig. S6:** Average nucleotide identity (ANIm) analysis of eleven *Eubacterium* strains. Strains are separated into three distinct clades comprising *E. limosum*, *E. callanderi*, and *E. maltosivorans*.


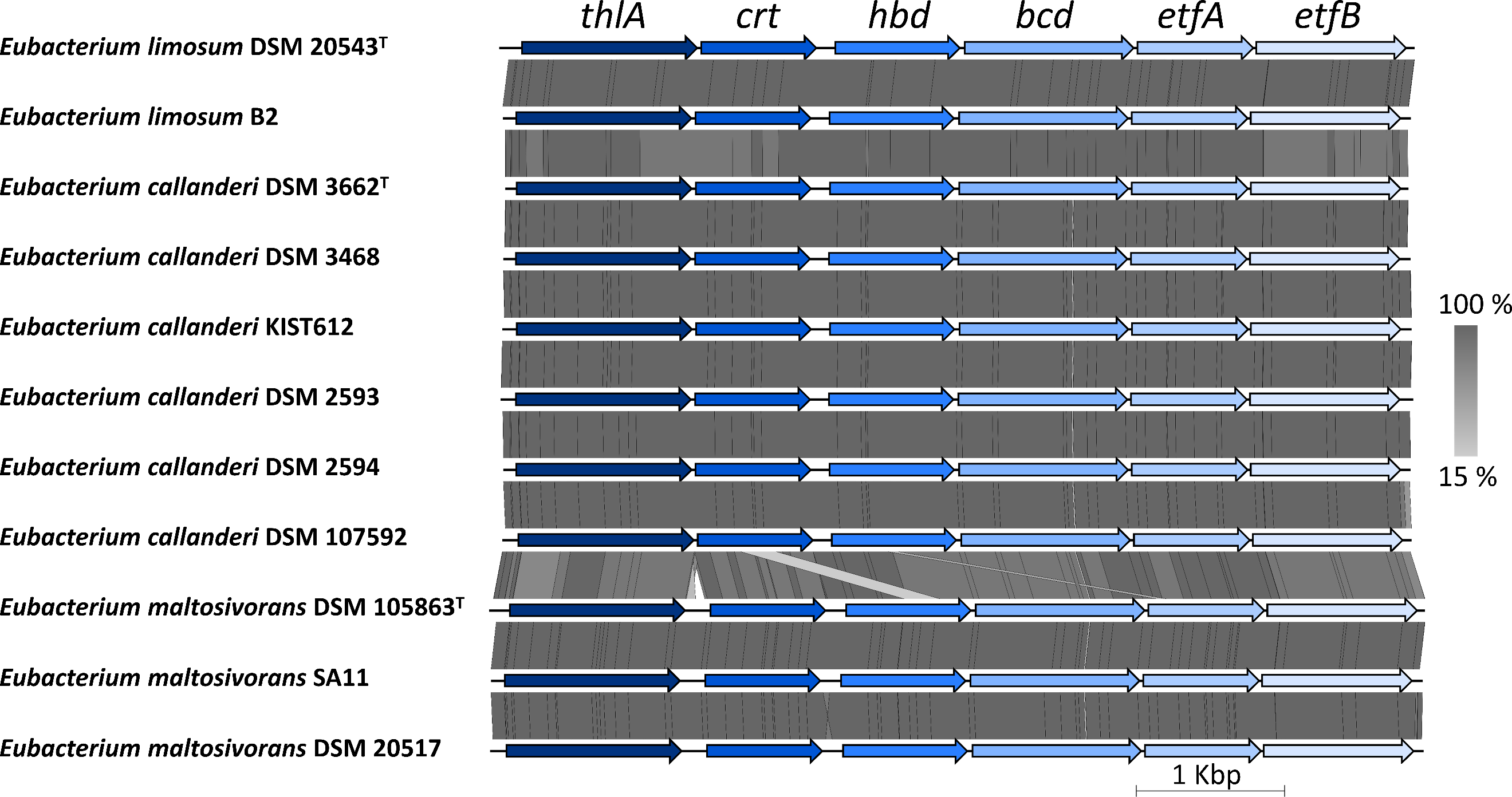
**Fig. S7:** Arrangement of *bcs*/*hcs* operon genes of the eleven analyzed *Eubacterium* strains
